# The Challenge of Time‐to‐Event Analysis for Multiple Events: A Guided Tour From Time‐to‐First‐Event to Recurrent Time‐to‐Event Analysis

**DOI:** 10.1002/bimj.70107

**Published:** 2026-01-28

**Authors:** Sandra Schmeller, Alexandra Erdmann, Jan Beyersmann, Christiane Angermann, Ann‐Kathrin Ozga

**Affiliations:** ^1^ Institute of Statistics Ulm University Ulm Germany; ^2^ Comprehensive Heart Failure Centre University and University Hospital Würzburg Würzburg Germany; ^3^ Department of Internal Medicine I University Hospital Würzburg Würzburg Germany; ^4^ Institute of Medical Biometry and Epidemiology University Medical Center Hamburg‐Eppendorf Hamburg Germany

**Keywords:** Markov assumption, method‐comparison, multistate model, recurrent events

## Abstract

Clinical trials often compare a treatment to a control group concerning multiple possible combined time‐to‐event endpoints like hospital‐free survival. Thereby, the first endpoint may occur more than once (“recurrent”), whereas the second endpoint is absorbing. Inclusion of all observed events in the analysis can increase the power and provide a more complete picture of the disease but it needs more sophisticated methodology. We give a stepwise guidance on how to extend the simple time‐to‐first event model to complex multistate methodology, where multiple events are incorporated. We thereby consider non‐ and semiparametric methods and show how they are related. Special attention is given to the prerequisites of the models, for example, the Markov property, and their interpretation. Due to novel results in non‐Markov models, the summary measurements: state occupation probability, mean number of hospitalizations, and average length of stay allow an easy interpretation of a treatment effect in non‐Markov models if the censoring is random. Partly conditional transition rates can be estimated instead of hazards. We investigate the difference between partly conditional transition rates and hazards and the impact of the random censoring condition in a simulation study. Furthermore, the simulation study considers the sensitivity of a Markov test. Different estimators are introduced, and their use is explained based on data from the randomized controlled Interdisciplinary Network Heart Failure trial, which investigated the effects of a nurse‐coordinated disease management program. The aim is to give an overview of existing methods, present the assumptions, and elaborate on the differences in interpretation.

## 1 Introduction

In clinical trials, the primary interest often lies in the comparison of a treatment to a control group regarding the occurrence of a specific event, for example, myocardial infarction, death, or hospitalization. Standard methods for analyzing such an endpoint include time‐to‐event models, such as the Kaplan–Meier method or the standard Cox proportional hazards model. These methods consider the time until the first event occurrence (Allignol et al. 2016; Angermann et al. 2012). Consequently, it is often overlooked that a patient may experience more than one nonfatal event, such as multiple hospitalizations. The longer the follow‐up, the more recurrent events are likely to occur. This multiple time‐to‐event scenario is also referred to as recurrent time‐to‐event. Hence, models for recurrent time‐to‐event analysis are of interest, as the inclusion of all observed events can increase power and provide a more complete picture of the disease (Ozga et al. 2018). Often used methods in such a setting include semiparametric Cox proportional hazards‐based models, for example, the model by Andersen and Gill, Prentice, Williams, and Peterson, as well as proportional rate models, for example, Ghosh and Lin (Andersen et al. (2019); Ghosh and Lin (2002), and Prentice et al. (1981)). Furthermore, a fatal event, such as death, may be observed and is either part of the primary endpoint or serves as a competing event for the primary event of interest (Furberg et al. 2022). If several event types are of interest, the so‐called composite endpoints can be considered. Moreover, multistate models offer an alternative to standard time‐to‐first event analysis in settings with multiple event occurrences. These models can be used to model several scenarios, like a so‐called illness–death model with recovery, where a patient can suffer several intermediate recurrent events before the terminal event happens. The transitions between the different events, for example, being alive at home to hospitalization or death, can be described by the so‐called transition hazards or probabilities. We illustrate this with a schematic display of the multistate model, showing which transitions are considered by semiparametric methods. There exist nonparametric estimators for the transition—hazards and—probabilities (Allignol et al. 2016; Andersen et al. 1993; Cook and Lawless 2022). However, these methods typically require the so‐called Markov assumption, which assumes that the occurrence of the next event is independent of previous events, given the current state. Rate‐based models exist where the assumption is relaxed. Furthermore, marginal probabilities, such as state occupation probabilities, the expected number of recurrent events (e.g., hospitalizations), and the average length of stay in the hospital allow for a causal interpretation of a treatment difference in a randomized trial, even in non‐Markov models with random censoring (Nießl et al. 2023). We emphasize the advantage of these quantities as they provide an easy interpretation with fewer model assumptions. The most significant restriction is that censoring must be random, that is, the event times and the censoring time must be stochastically independent. This stochastic independence is often true if the censoring is only related to external factors and not to other events, as in event‐driven trials. The latter is a special example of independent censoring.

This work aims to give step‐by‐step guidance for the applied researcher on how standard time‐to‐first event analysis can be extended to multiple time‐to‐event analysis, giving special attention to the underlying model assumptions. Therefore, the methods are described in detail, including their assumptions, and are illustrated and compared using a real data example. Two types of aftercare for patients with systolic heart failure are compared in a randomized clinical trial from the Interdisciplinary Network for Heart Failure. The events of interest are recurrent hospitalization and the competing event of death. Based on this, a simulation study provides more insights into possible errors when the model assumptions are not fulfilled.

In Section 2, a real data example is introduced, along with an explanation of the underlying medical question. We begin with the definition of the time‐to‐first event methodology in Section 3. Section 4 introduces multistate models, especially the illness–death model and the progressive model. The link between semi‐ and nonparametric methods is explained, and we suggest some marginal quantities for measuring a treatment difference. The analysis of the data example is shown in Section 5. The simulation study focuses on the Markov assumption and random censoring (Section 6). We finish with a concluding discussion in Section 7.

## 2 Case Study

A previous analysis from Angermann et al. (2012) considers a multicenter randomized controlled trial with 715 patients hospitalized with acute heart failure (HF), to either undergo 18 months of nurse‐coordinated remote patient management (HeartNetCare‐HF [HNC]) and usual care (UC) or UC alone (HNC+UC: *n* = 352; UC: *n* = 363). Patients who had at discharge an ejection fraction of 40% or less and provided written informed consent were eligible. During 180 days of follow‐up, the primary endpoint, a composite of time to all‐cause death or hospitalization, was neutral (hazard ratio 1.02, 95% confidence interval [CI]: 0.81–1.30; *p*‐value = 0.890, HNC+UC: 130 primary outcome events; UC 137 primary outcome events). The Extended Interdisciplinary Network Heart Failure (E‐INH) trial randomized 1022 patients using the same inclusion criteria and primary outcome (HNC+UC: 509 patients; UC: 513 patients) (Angermann et al. 2023). Clinical outcome events were recorded through 60 months (including 42 months of intervention‐free follow‐up). The median follow‐up time was 2596 days, and 663 deaths and 3016 rehospitalizations (with a maximum of 27 events per patient) occurred. Using the conventional time‐to‐first‐event approach, both repeated rehospitalizations and fatal events that occurred after the first rehospitalization are neglected. We aim to include all fatal events and the total number of rehospitalizations to facilitate a more comprehensive evaluation of the possible benefits of the intervention. Censoring occurs due to study closure or lost to follow‐up without a systematic reason. We, therefore, assume censoring to be essentially random, which means that the censoring time is stochastically independent of the time‐to‐event process (see Section 3). The methods described in Sections 3 and 4 are illustrated using this E‐INH data example; however, they can also be applied to other recurrent event settings (Erdmann et al. 2024).

## 3 Time‐to‐First Event Methodology

Standard survival and event history analysis considers the time‐to‐first occurrence of a specific type of event, for example, time to all‐cause death, for an individual. However, different event types are often of interest, such as hospitalization or death. In such a case, an analysis approach might be the so‐called composite endpoint, where the time to the first occurring event, for example, hospitalization or death, whatever comes first, is considered.

Another extension of the standard survival model is the so‐called competing risks. Thereby, the time until the first event is considered, but it is distinguished by the type of event, for example, first hospitalization and death before hospitalization. This model provides a deeper understanding of the risk of the first event. The occurrence of an event can be described using the so‐called hazard or hazard function. In the context of different event types, the cause‐specific hazards are described, that is, each event type has its own hazard. The cause‐specific hazard $\lambda_{0j}\left(t\right)$, multiplied by a short time interval, dt, is the instantaneous probability of having an event of type ε=j,j∈{1,…,J} with event time T given previous survival:

(1) $$\begin{array}{c} \lambda_{0j}\left(t\right)dt=P\left(T\in \left[t,t+dt\right),\varepsilon =j|T\geq t\right). \end{array}$$

Thereby, J is the number of different event types. The notation 0j describes the transition from the initial state 0 to the event (or state) j. The all‐cause hazard is $\lambda \left(t\right)=\sum_{j}^{J}\lambda_{0j}\left(t\right)$. The cumulative hazards are $\Lambda_{0j}\left(t\right)=\int_{0}^{t}\lambda_{0j}\left(u\right)du$. One must use more advanced estimation procedures because our data are right‐censored. Often, random censoring is assumed, that is, T and the censoring time C are stochastically independent, possibly given covariates. However, survival methodology allows for more general “independent censoring,” for example, event‐driven trials (Rühl et al. 2023); here, the assumption is that the additional knowledge does not alter event intensities, as defined precisely by Aalen et al. (2008). The distinction will later be of relevance for recurrent events methodology. The basic nonparametric estimation of $\Lambda_{0j}\left(t\right)$ is defined via the Nelson–Aalen estimator:

(2) $$\begin{array}{cc} {\hat{\Lambda }}_{0j}\left(t\right) & =\sum_{k=1}^{K}\frac{\text{Number of observed type}\;j\;\text{transitions/events at}\;t_{k}}{\text{Number of patients at risk just prior to}\;t_{k}}, \end{array}$$

with $0<t_{1}<t_{2}<\cdots \;<t_{k}=t$ the ordered sequence of observed failure/event times. The standard regression approach is the semiparametric Cox proportional hazards model:

(3) $$\begin{array}{c} \lambda_{0j}\left(t|Z\right)=\lambda_{0j}^{0}\left(t\right)\exp\left(\beta_{j}Z\right), \end{array}$$

where $\lambda_{0j}^{0}\left(t\right)$ refers to a common (cause‐specific) baseline hazard, that is, the instantaneous baseline risk for an individual to experience the event (e.g., first hospitalization) at time t (given no prior event). Furthermore, in Equation (3), it will be assumed that a comparison of two treatment groups in a clinical trial shall be considered; hence, Z = 0 is defined for an individual in the control group and Z=1 for an individual in the intervention group.

Probabilities are a function of all cause‐specific hazards. In the standard survival setting, the probability of the event is

$$\begin{array}{c} F\left(t\right)=1-S\left(t\right)=P\left(T\leq t\right)=1-\exp(-\int_{0}^{t}\lambda \left(u\right)du), \end{array}$$

which can be estimated with the nonparametric Kaplan–Meier estimator for the survival function S(t). With competing risks, the cumulative incidence of suffering an event of type ε=1 is

$$\begin{array}{c} P\left(T\leq t,\varepsilon =1\right)=\int_{0}^{t}P\left(T>u-\right)\lambda_{01}\left(u\right)du, \end{array}$$

where P(T>u−)=S(u−). Since the all‐cause hazard is a part of the survival function, the probability of an event of type 1 depends on all cause‐specific hazards. $\hat{P}\left(T>u-\right)$ can be estimated with the Kaplan–Meier estimator and ${\hat{\lambda }}_{01}\left(u\right)$ with the increment of the Nelson–Aalen estimator. Alternatively, the cumulative incidence can be estimated with the Aalen–Johansen estimator, which we will introduce in the next Section (Beyersmann et al. 2011). Regression models for the cumulative incidence are not straightforward because both event‐specific hazards influence the probability of an event occurring. The Fine and Gray model uses a subdistribution process by setting the competing event times to infinity and estimates the Cox‐regression model for the event of interest. Inverse probability of censoring weights are used to address with the unknown censoring times of the competing event and, consequently, the unknown risk set. Alternatives are the proportional odds model or regression with pseudo‐values (Beyersmann et al. 2011; Eriksson et al. 2015; Klein and Andersen 2005).

We will be interested in the effect of the randomized treatment intention. This has recently been challenged when expressed on the hazard scale because the hazard definition, as in Equation (1), conditions on a post‐randomization event (Martinussen 2022). We consider interventional effects on hazard functions, such as $t\mapsto \lambda_{0j}\left(t\right)$, t≥0, j=1,…,J, which can be transformed into unconditional probabilities as above. The idea is that an intervention can now change a population's future hazard or intensity functions, which translates into an interventional effect on probabilities. (See Beyersmann et al. 2025 for an in‐depth discussion.) Later, we consider further easy‐to‐interpret marginal quantities in a recurrent events setting.

## 4 Recurrent Events and Multistate Models

In this section, we gradually extend the competing risks model to multistate models. We begin with the smallest three‐state model, the illness–death model, and then extend it to a progressive model. A special focus lies in explaining what the Markov assumption means in the models. The last section shows what can still be estimated consistently if this assumption is not met.

### 4.1 Illness–Death Model With Recovery

**FIGURE 1:**
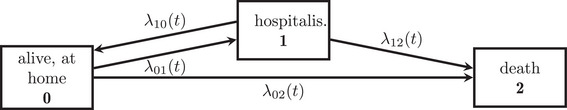
Illness–death model with recovery. The states are represented with boxes. Possible transitions between the states are marked with arrows. Hospitalis.: hospitalization.

Since we aim to analyze all events observed for an individual and describe the entire disease process, we extend the competing risk setting in Section 3 to consider several intermediate events (hospitalizations and discharges alive) and a death event that occurs after one or more intermediate events. Thereby, the initial state is alive and at home. Afterward, a patient can move to one of the states, undergo hospitalization, or die. Reaching the hospitalization state, one can die or be discharged from the hospital. Being discharged, the patients are again in state 0, the initial state, and can suffer both possible events—hospitalization and death—again. The situation described can be represented schematically by an illness–death model with recovery (see Figure 1).

The transitions between the different states are marked with arrows in Figure 1. For every transition, one can estimate a transition hazard. Therefore, possible hazards are $\lambda_{01}\left(t\right),\lambda_{02}\left(t\right),\lambda_{10}\left(t\right)$, and $\lambda_{12}\left(t\right)$. In Figure 1, we have multiple event states one can be in, but the number of states is limited. This is referred to as a multistate process with a finite state space. Let ${\left(X_{t}\right)}_{t\geq 0}$ be this multistate process with finite state space {0,1,2}, denoting the state where an individual i is in at time t. Let $X_{t}$ be adapted to its self‐exciting filtration, practically speaking, the history of the multistate process up to time t, $F_{t-}$. Then, the transition hazard from state h to j is defined via

(4) $$\begin{array}{c} \lambda_{hj}\left(t;F_{t-}\right)dt=P\left(X_{\left(t+dt\right)}=j|X_{t-}=h,F_{t-}\right), \end{array}$$

with j=0,1,2 and h<j or h=1 and j=0. Our data are right‐censored, and the independent censoring assumption states that the additional knowledge of the censoring process does not influence the event intensities. Therefore, the event intensities that are dependent on the entire past are equal to depend on the observed past. The observed past contains previous states and previous transition time‐points as well as the censoring time C. It is not possible to test the censoring scheme. One must judge it with the help of the medical background. Furthermore, we assume that the multistate process $X_{t}$ is Markov. That means that the condition on the previous state is equal to the condition on the entire past, that is, the Markov assumption means that the transition hazard does not depend on previous states, the number of previous states, nor on previous event times like the entry time into the state h. The dependence on (the number of) previous states or the entry time into the current state can be tested, for example, by including the number of previous states in a regression model as a covariate. We will investigate this in the simulation section. The transition hazard in a Markov multistate model is

(5) $$\begin{array}{c} \lambda_{hj}\left(t\right)dt=P\left(X_{\left(t+dt\right)}=j|X_{t-}=h\right). \end{array}$$

The corresponding cumulative hazard can be estimated nonparametrically using the Nelson–Aalen estimator (2). The indices in (2) “0j” should be adapted to “hj” for the transition from state h to state j. The risk set comprises patients who are in state h at different time points. Alternatively, the transition hazards can be estimated using a semiparametric Cox model. For $\lambda_{01}\left(t\right)$, this Cox‐type model is called the cause‐specific Andersen–Gill model (AG) under a Markov assumption. A patient contributes to the estimation of the $\lambda_{01}\left(t\right)$ hazard only if they are in state 0, but a patient can drop in and out of the risk set of the nonfatal states 0 and 1. The AG model does not distinguish between the types of events (first/ second/ further hospitalization) because the transition‐specific hazard $\lambda_{01}\left(t\right)$ is assumed to depend on time (through the baseline hazard) and the treatment group, which is known at study start (Ozga et al. 2018). That the hazard is independent of the history otherwise, it is often violated in real datasets, as seen, for example, in Ozga et al. (2018). They call the estimate from this model “mixed effect” because it is a combination of the effect of the treatment and the effect due to previous events. To overcome this, one might think of including the number of previous events as a covariate in the model. However, suppose the corresponding effect shows a relevant influence. In that case, it is, first, a hint that the Markov assumption is violated, and, second, it adjusts for post‐randomization events influenced by the treatment (Furberg et al. 2022).

In the illness–death model, we are also interested in the transition probabilities. Here, any transition probability depends on all transition hazards. Within the illness–death model with recovery, we have no simple formula consisting of survival functions and hazards for the transition probability $P_{hj}\left(s,t\right)=P\left(X_{t}=j|X_{s}=h\right)$ as in the competing risks setting. However, the general nonparametric Aalen–Johansen estimator can still be obtained as a function of the matrix of Nelson–Aalen estimators. It is the matrix product over all event times u, u∈(s,t] of

$$\begin{array}{c} \hat{P}\left(s,t\right)=\prod_{u\in (s,t]}\left(I+\Delta \hat{\Lambda }\left(u\right)\right), \end{array}$$

with $\hat{P}\left(s,t\right)$ the matrix of probability estimates with (h,j)th entry ${\hat{P}}_{h,j}\left(s,t\right)$, I the identity matrix, and Λ(u) for the matrix of Nelson–Aalen estimators with (h,j)th entry ${\hat{\Lambda }}_{h,j}\left(u\right)$ as in (2) and diagonal entries ${\hat{\Lambda }}_{hh}\left(u\right)=-\sum_{j,j\neq h}{\hat{\Lambda }}_{hj}\left(u\right)$. The increment $\Delta \hat{\Lambda }\left(u\right)$ is the matrix with entries ${\hat{\Lambda }}_{hj}\left(u\right)-{\hat{\Lambda }}_{hj}\left(u-\right)$.

However, the Markov assumption, which is essential for hazard estimation and transition probabilities, which condition on a time s>0, can be violated. If we estimate (5) in a non‐Markov model subject to random censoring only, we estimate partly conditional transition rates. We will explain in Section 4.3 which quantities can also be estimated in non‐Markov models with known estimators. In the next step, we extend the illness–death model to a more complex multistate model.

### 4.2 An Extended Multistate Model

**FIGURE 2:**
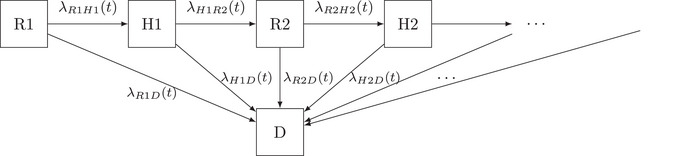
Extended multistate model. The states are represented with boxes. R1, R2, …: at risk and at home, H1, H2,…: in hospital, D: death. Possible transitions between the states are marked with arrows.

In this section, we demonstrate how to extend the illness–death model to account for the number of recurrent events, thereby making the Markov property more plausible. The schematic display for the corresponding event process is given in Figure 2. Thereby, each arrow models a transition. The difference from Figure 1 is that the hazards depend on the number of intermediate events (at home and in the hospital). That means we model different hazards for every possible transition: first time at home to first time in hospital and first time at home to death, first time in hospital to second time at home, and first time in hospital to death, etc.

The cumulative hazard and transition probabilities can also be estimated with the Nelson–Aalen estimator and the Aalen–Johansen estimator. In contrast to the illness–death model, one has to specify the maximum number of transitions ahead. Nevertheless, the Markov assumption is less restrictive in this case. The Markov assumption assumes that the hazard only depends on the current state and time. The implication is that the current transition hazard may depend on the previous number of recurrent events. This is in contrast to the illness–death model with recovery, where such a dependence is not modeled (Andersen and Ravn 2023). In many applications like ours, this model may be more realistic. However, one needs a sufficiently large number of patients and transitions for plausible estimates of transition probabilities. Furthermore, in many applications, it might not be interesting to estimate a specific transition probability. Instead, marginal quantities might be more interesting, for example, the mean number of hospitalizations and the average length of stay. The following section introduces such features. For semiparametric treatment estimation, one can use the model by Prentice–Williams–Peterson (PWP), which is an extension of the AG model by additionally stratifying according to the number of previous events (e.g., hospitalizations). The model assumes a common treatment effect between each transition from home to hospital but allows transition‐specific baseline hazards, that is, in our model in Figure 2, the hazards $\lambda_{R1H1}\left(t\right),\lambda_{R2H2}\left(t\right),\lambda_{R3H3}\left(t\right),\text{…}$ in the Cox‐type form are different through the baseline hazard but share a common treatment effect. The PWP model compares patients who are at the same length since randomization and have the same history of the number of previous events (hospitalizations) (Kalbfleisch and Prentice 2011; Ozga et al. 2018).

### 4.3 Marginal Features for Multistate Models With Recurrent Events

As described in the previous sections, the estimation of transition hazards and transition probabilities is usually based on the Markov assumption. To overcome this, partly conditional transition rates can be used, which do not require the Markov assumption, but their estimation relies on random censoring (Nießl et al. 2023). Random censoring cannot be tested in the data and must be justified with the medical background and data generation mechanism. In this section, we introduce marginal estimated quantities and estimators that do not require the Markov assumption and complete the analysis with partly conditional transition rates. That is, we assume that $\lambda_{hj}\left(t\right)$ in Equation (4) does depend on $F_{t-}$, and rates are partly conditional in that they only condition on $X_{t-}$ but not on $F_{t-}$ (see Equation 5). The cumulative partly conditional rate can be estimated with the Nelson–Aalen estimator (Nießl et al. 2023). We will demonstrate in Section 5 that random censoring is a crucial assumption for valid estimation.

The rate‐based equivalent to the AG model (not requiring the Markov assumption) is the rate model proposed by Ghosh and Lin (2002). The rate function conditional on Z is defined by

$$\begin{array}{c} d\mu_{Z}\left(t\right)=E\left(dN^{R}\left(t\right)|Z\right), \end{array}$$

where $N^{R}\left(t\right)$ is the uncensored number of recurrent events in [0,t].

The interpretation in terms of the model in Figure 2 is that, for example, $N^{R}\left(t\right)=2$ for the recurrent event hospitalization if and only if a transition into H2 has been observed on [0,t], and no transition into H3 has been observed on [0,t]. Here, durations in hospitals are assumed to be negligible in comparison to stays at home (Lin 1994). Nevertheless, the concept of the rate here considers each transition from home to the hospital (also from R1 to H1). The interpretation of $d\mu_{Z}\left(t\right)$ is as follows:

$$\begin{array}{cc} \mu_{Z}\left(t\right) & =E(N^{R}\left(t\right)|Z)\\ & =\int_{0}^{t}S\left(u-|Z\right)\cdot dR\left(u|Z\right), \end{array}$$

where S(u) is again the survival function and dR(u|Z) is the expectation of $dN^{R}\left(u\right)$ given survival prior to u and given Z. In this sense, dR(u|Z) averages over individual recurrent event intensities, where the latter do not only condition on Z and prior survival, but on the individual's entire past (Andersen et al. 2019). The Ghosh and Lin model defines $d\mu_{Z}\left(t\right)$ in a Cox‐type form. In the estimation procedure, the baseline rate and the regression coefficient can be estimated using a Breslow‐type estimator and a score function similar to those of the Cox model, provided that the censoring times are known. This is the case, for example, if censoring occurs only due to study closure. Otherwise, the inverse probability of censoring (or survival) weighting technique can be used, analogous to the Fine and Gray model, to estimate the risk set (Beyersmann et al. 2011; Ghosh and Lin 2002; Martinussen and Scheike 2006). In cases where the weights have large values, Curtis et al. (2007) suggest trimming these weights or analyzing subgroups. Alternatively, the weighted analysis can be replaced by multiply imputing the censoring times (Ruan and Gray 2008; Schaubel and Zhang 2010).

Furthermore, marginal features such as state occupation probabilities, the mean number of recurrent hospitalizations, or the average length of stay in the hospital are attractive because their interpretation is more straightforward than that of intensities or rates. Also, there is no discussion that a contrast of such features, being marginal, has a causal interpretation. The state occupation probabilities in an illness–death multistate model are $Q_{l}\left(t\right)=P\left(X_{t}=l\right)=P\left(X_{t}=l|X_{0}=0\right)$ (all patients start in state 0 at time 0). The Aalen–Johansen estimator provides estimates for marginal state occupation probabilities, which offer a more comprehensive view of the distribution of event counts over time. Nießl et al. (2023) show that the Aalen–Johansen estimator remains a consistent estimator in a non‐Markov multistate model when censoring is random. The result for the Aalen–Johansen estimator is based on the proof that the Nelson–Aalen estimator is a consistent estimator for the cumulative partly conditional transition rates (as the Aalen–Johansen estimator is a transformation of the Nelson–Aalen estimator). The random censoring assumption ensures that the individual hazards in a non‐Markov model are independent and identically distributed (i.i.d.) random variables, and their average approaches the partly conditional transition rate.

With the state occupation probability, one can estimate the average length of stay in the hospital up to time t with

(6) $$\begin{array}{c} \int_{0}^{t}E\left(1\left(X_{u}=1\right)\right)du=\int_{0}^{t}Q_{1}\left(u\right)du \end{array}$$

and the mean number of hospitalizations (Erdmann et al. 2024). The commonly used marginal mean, exploiting all transition rates in the illness–death model, is defined as

(7) $$\begin{array}{c} \mu \left(t\right)=E\left(N^{R}\left(t\right)\right)=\int_{0}^{t}Q_{0}\left(u\right)\lambda_{01}\left(u\right)du. \end{array}$$

We estimate the marginal mean number of hospitalizations in the illness–death model, that is, the expected number to reach state 1 (see Figure 1). The used estimator is $\hat{\mu }\left(t\right)=\sum_{u_{k}\leq t}\hat{P}\left(X_{u_{k}}=0\right)\cdot \Delta {\hat{\Lambda }}_{01}\left(u_{k}\right)$, where $u_{k}$, k∈{1,…,K} are the ordered event times of a recurrent or terminating event. The state occupation probability $P(X_{u_{k}}=0)$ can be estimated with the Aalen–Johansen estimator and the cumulative partly conditional transition rate, or cumulative hazard in a Markov model with the Nelson–Aalen estimator. Erdmann et al. (2024) describe different estimators for the marginal mean, which is also based on an underlying progressive multistate model, as shown in Figure 2, allowing transition hazards/partly conditional rates to be dependent on previous recurrent events. They found that the estimators perform comparably, provided that the requirements are fulfilled (e.g., random censoring in non‐Markov models). As argued by Ghosh and Lin (2002) and Andersen et al. (2019), the marginal mean and the average length of stay of two groups (e.g., treatment groups) should not be considered alone. A reduction of the mean number and, therefore, a supposed protective effect of the therapy can result either because of a reduced event intensity for the recurrent event or because the patients stay longer in the hospital, which can be evaluated with the average length of stay. However, an increased mortality can also lead to a reduced number of hospitalizations and possibly shorter average length of stay in the hospital.

Wei et al. (2023) suggest quantifying the relation between hospitalization and death with a nonparametric summary measure to compare two treatment groups. The quotient of the expected number of hospitalizations per group divided by the overall survival classifies the number of hospitalizations in relation to the survival:

(8) $$\begin{array}{c} RR\left(t\right)=\frac{E\left(N^{trt}\left(t\right)\right)/\int_{0}^{t}1-Q_{2}^{trt}\left(u\right)du}{E\left(N^{uc}\left(t\right)\right)/\int_{0}^{t}1-Q_{2}^{uc}\left(u\right)du} \end{array}$$

with $N^{trt}\left(t\right)$ and $N^{uc}\left(t\right)$ the number of hospitalizations for each treatment group, superscript trt for treatment, and superscript uc for usual care, respectively, and $Q_{2}^{k}\left(t\right)$ is the state occupation probability of state 2, which is one minus the survival probability, for each treatment group k,k∈{0,1}. The interpretation from a patient perspective is “How many hospitalizations I can expect, divided by how long I can expect to live in the next t years” (Wei et al. 2023). This is set in relation to the other treatment group. A value below 1 indicates that the expected number of hospitalizations per unit time alive is reduced (by 1− the value %) in the treatment group compared to the ones in the usual care group. Wei et al. (2023) suggest interpreting the estimates (RR(t)) in relation to other estimates with changing populations and time periods t. Uncertainty can be quantified with bootstrapped confidence intervals (Wei et al. 2023).

## 5 Results of the Application

The presented methods are applied to the data from the E‐INH (see Section 2). We use the randomly right‐censored follow‐up data and start with the time‐to‐first‐event model. The aim is to gradually extend the models to obtain precise estimates of a treatment difference concerning first, recurrent hospitalization, and death. We will show that non‐ and semiparametric methods complement each other. First, a treatment difference is investigated in terms of overall survival, hospital‐free survival, and the competing risks setting, with first hospitalization and death without previous hospitalization as competing events. Second, multistate models are introduced to consider not only the first observed event but also subsequent events. The totality of the different non‐ and semiparametric methods, as well as marginal approaches, can display a complete picture of the multistate process, which leads to more insights into how the investigative treatment works. However, some of these models typically require further assumptions, which we are hence considering in our analysis.

The treatment shows no harmful effect on survival. Rather, Kaplan–Meier curves (see the Supporting Information) and the estimated hazard ratio of the Cox model (hazard ratio [HR]: 0.820, *p* = 0.011, CI: [0.70,0.96], see Table 1) provide a signal for a beneficial effect of the treatment group. Including the first hospitalization, the favorable trend persists for hospital‐free survival (HR: 0.904, *p* = 0.133, CI: [0.79,1.03], see Table 1 and Figure S2 in the Supporting Information).

**TABLE 1:** Results from the semiparametric models. The combined endpoint is hospitalization or death. HR: hazard ratio, RR: rate ratio, *p*: *p*‐value, 95% CI: 95%‐confidence interval (In AG: estimated with robust standard errors), trt.: treatment, OS: overall survival, hosp.: hospitalization

**Cox model**									
**Endpoint:**	**Overall survival**								
	HR	*p*	95% CI						
trt. vs. usual care	0.820	0.011	[0.70,0.96]						
**Endpoint:**	**Hospital‐free survival**			**Hospitalization**			**Death before hosp**.		
	HR	*p*	95% CI	HR	*p*	95% CI	HR	*p*	95% CI
trt. vs. usual care	0.904	0.133	[0.79,1.03]	0.872	0.060	[0.76,1.01]	1.125	0.516	[0.79,1.60]
**AG model**									
**Endpoint:**				**Hospitalization**					
				HR	*p*	95% CI			
trt. vs. usual care				0.861	0.053	[0.74,1.00]			
trt. vs. usual care				0.878	0.018	[0.79,0.98]			
previous hosp.				1.240	<0.001	[1.21,1.27]			
**Ghosh and Lin model**									
**Endpoint:**				**Hospitalization**					
				RR	*p*	95% CI			
trt. vs. usual care				0.860	0.059	[0.74,1.01]			
**PWP model**									
**Endpoint:**				**Hospitalization**					
				HR	*p*	95% CI			
trt. vs. usual care				0.881	0.008	[0.80,0.97]			
trt. vs. usual care				0.908	0.021	[0.84,0.986]			
entry time				1.002	<0.001	[1.0014,1.0018]			

**FIGURE 3:**
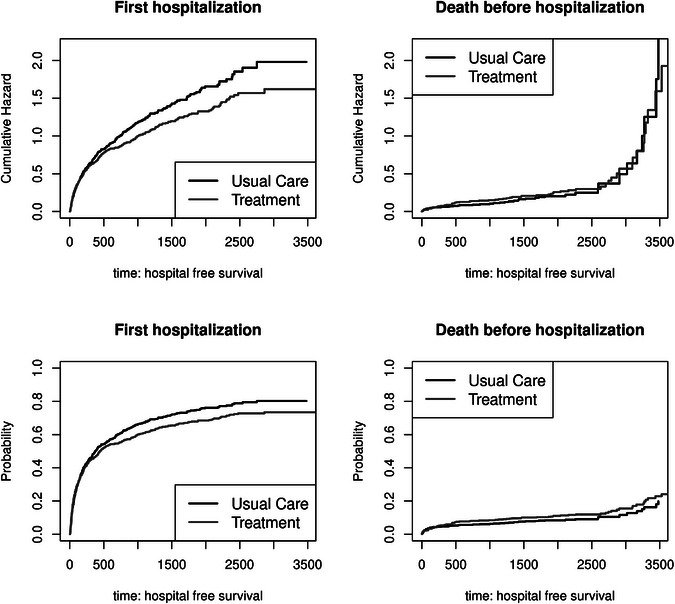
Competing risks setting: time‐to‐first event analysis with competing endpoints, hospital admission, and death before hospital admission. Upper panel: Nelson–Aalen estimates of the cumulative hazard; lower panel: Aalen–Johansen estimates of the cumulative incidence.

We extend this analysis and differentiate the endpoints first hospitalization and death before hospital admission in a competing risk setting to determine if treatment reduces the risk of first hospitalization, death before hospital admission, or both. Figure 3 (first row) shows the plots of the estimated cumulative hazards. The hazard for the first hospitalization is higher in the usual care group. We see no visual difference for the death hazard. Treatment works by reducing the first hospitalization, which is in line with the results from the Cox model (Table 1). Considering the probability, we can also observe a more substantial increase in the probability of first hospitalization of the usual care group. Hence, patients in the treatment group have a delayed occurrence of a first admission to the hospital (reduced cumulative hazard and incidence probability in Figure 3, first column). The probability of death (w/o prior hospitalization) seems to be slightly higher in the treatment group, although we cannot observe a visual difference between the hazards in Figure 3. The competing risk analysis disentangles the effects leading to the protective point estimate for treatment on hospital‐free survival, but it must not be interpreted as indicating that treatment is harmful concerning survival. Figure 3 displays the estimated cumulative event probabilities for the first hospitalization and death before the first hospitalization, respectively. The estimated probability of hospitalization is lower for treatment. Because these probabilities must eventually add up to one, we would need to expect eventually more deaths before hospitalization in the treatment group. There is a slight indication of this in Figure 3 (bottom right). The top panel of Figure 3 illustrates the Nelson–Aalen estimates, which help to interpret the event‐specific hazard ratios: The hazard of first hospitalization is the major hazard for around the first 2000 days, and treatment appears to reduce the (cumulative) hazard, while a difference is hardly visible in Figure 3, top right. This is particularly in line with the confidence intervals presented in Table 1.

To investigate how the treatment works more precisely, we also include recurrent hospitalizations and death events after hospitalization. We begin with an illness–death model with recovery and first assume that the model is Markov.

**FIGURE 4:**
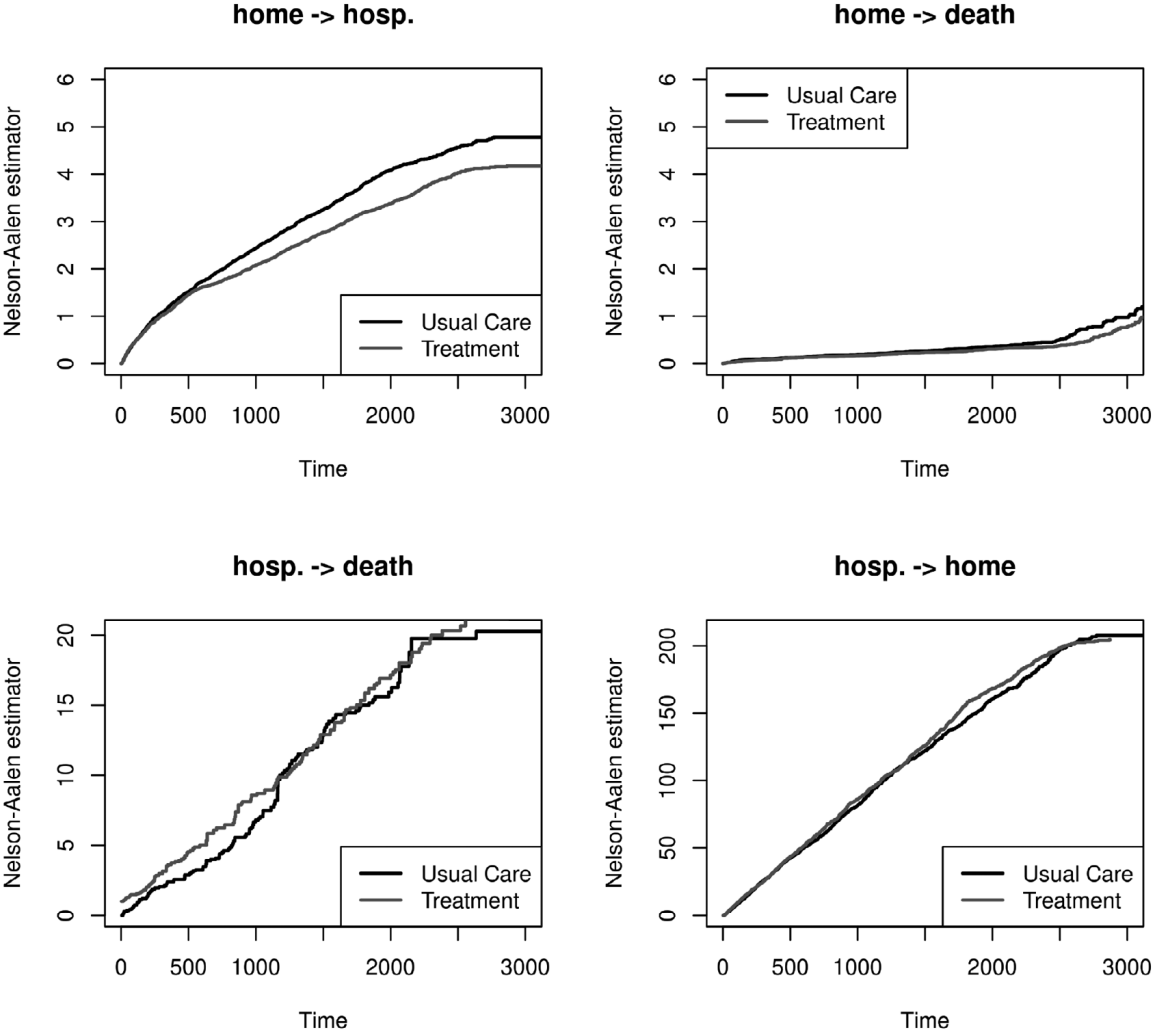
Nelson–Aalen estimates in the illness–death model with recovery; note the different scale levels. hosp.: hospitalization

The Nelson–Aalen estimates are shown in Figure 4. We observe a higher cumulative hospitalization hazard of the usual care group and no visual difference for the death at home. The scale level for the other two transitions, from hospital to death and from hospital to home, is different from those out of the state “at home” in the Figure, indicating very high hazards in comparison. Therefore, it is very likely to get out of the hospital, and dying in the hospital has a more pronounced hazard than at home. However, we cannot see an apparent visual treatment effect here.

Even in cases where the Markov assumption does not hold, Figure 4 shows meaningful estimates of cumulative partly conditional transition rates because the underlying censoring is assumed to be random.

A semiparametric method for quantifying the instantaneous risk contrast for the transition from home to hospital is the cause‐specific AG model for the event hospitalization. During the stay in the hospital, the patients are not at risk for further hospital admission. Therefore, the analysis does not consider hospitalized patients at risk for hospitalization (cf. Figure 1). The protective effect that treatment reduces the cumulative hospitalization hazard rate can be confirmed with comparable HRs in the AG model (for recurrent hospitalization) and the Cox model in the competing risks setting (for first hospitalization). The assumption of this model (a Markovian illness–death model) is that the hazard only depends on time but not on the number of previous events. To test this, we include the number of previous events as a covariable, which detects a clear violation of the Markov property with respect to hospitalization (see Table 1). Hence, the marginal model for the rate counting the hospitalizations, the Ghosh and Lin model, is calculated and shows comparable results to the AG model. Results are arguably comparable because of the low mortality that is similar between groups.

**FIGURE 5:**
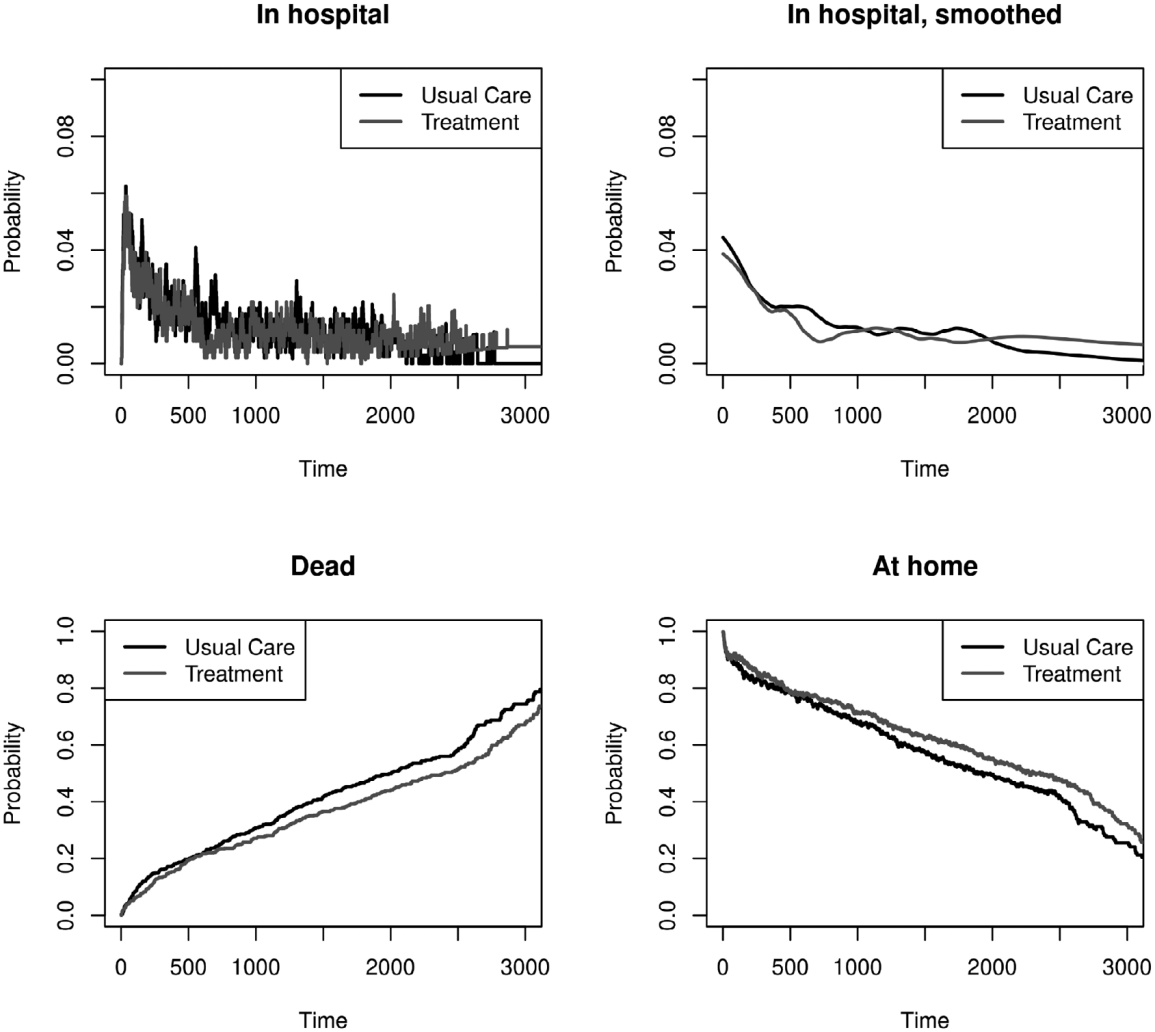
State occupation probabilities in an illness–death model with recovery.

The Nelson–Aalen plots (see Figure 4) imply that treatment should lead to a reduced probability of hospitalization. In a Markov illness–death model, transition probabilities can be estimated with the Aalen–Johansen estimator. In non‐Markov models and the presence of random censoring, the Aalen–Johansen estimator of the state occupation probabilities is still consistent (Nießl et al. 2023) and shown in Figure 5. The first plot in Figure 5 shows the occupation probability of being in the hospital, but it is difficult to read as it fluctuates very strongly. It implies that moving in and out of the hospital is common. The very high rates out of state 1 (see Figure 4) support that moving out of the hospital is common. The second plot in Figure 5 shows smoothed versions of these probabilities (loess with span parameter set to 25%) (Cleveland et al. 1992). However, the smoothed plot does not truly clarify the picture at hand. Hence, it is helpful to estimate the average length of stay and the mean number of hospitalizations for a better interpretation.

**FIGURE 6:**
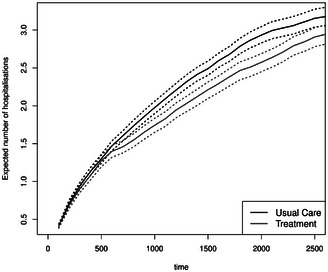
Expected number of hospitalizations with bootstrapped 95% confidence intervals.

**FIGURE 7:**
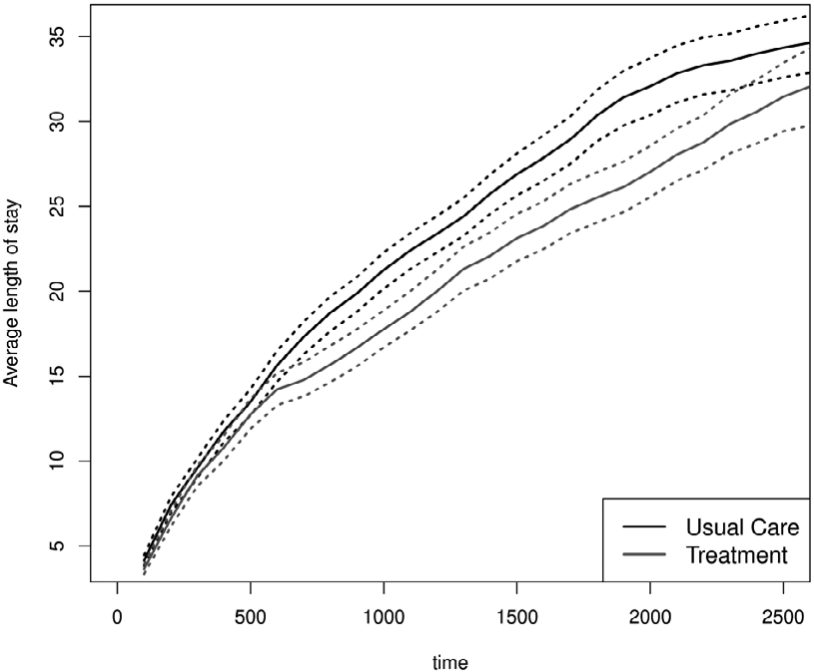
Average length of stay in the hospital with bootstrapped 95% confidence intervals.

**FIGURE 8:**
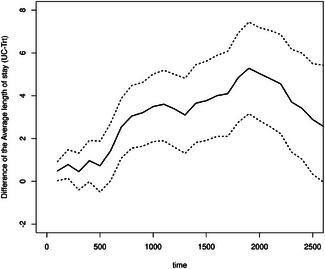
Difference of the average length of stay in the hospital from the usual care group (UC) minus the treatment group (Trt) with bootstrapped 95% confidence intervals.

The mean number of hospitalizations is reduced for the treatment group from day 800 onward (see Figure 6). This is in line with the reduced hazard from home to hospital in the illness–death model. As the mortality hazard and probability (Figure 5, right) are not higher in this group, a reduction in the mean number of hospitalizations is beneficial as it does not result from more deaths. Furthermore, the average length of stay in the hospital is longer for the usual care group between Day 700 and Day 2500 (Figure 7). This quantity can be more easily interpreted than the state occupation probability of being in the hospital. Please note that we plot Figures 6, 7, 8 until day 2500 because little happens afterward (see the first left Figure 5). The bootstrapped 95% confidence intervals are the basic bootstrap confidence limits of Davison and Hinkley (1997, 194) and are computed with 1000 bootstrap samples. The other two state occupation probabilities (in Figure 5) show the probability of dying, one minus the overall survival probability, and the probability of being at home. The figures confirm the previous results, indicating a slight improvement in survival for the treatment group (as shown by the Cox model; see Table 1) and a higher likelihood of being at home from day 500 onward for those receiving the treatment. To put the mean number of hospitalizations in relation to the survival probability, we calculate the ratio of the two groups of the two quantities until time t for four time points in days: t=500 and RR=1.1660, t=1000 and RR=0.9923, t=1500 and RR=1.0061, and t=2000 and RR=0.9990. All estimates are near 1.

An alternative to the present analysis is to relax the Markov assumption and distinguish between the number of recurrent events in the progressive model 2. We do not present figures here (see Supporting Information), which are generally in line with the presented results but are hampered by fewer events for later transitions. Within this framework, PWP is the analog of AG, and previous results are confirmed (see Table 1). In particular, we again find a violation of the Markov property when we include the entry time (in days) in the PWP model to test the Markov assumption. The HR near one result because of the entry time in days, the *p*‐value shows the significant influence of the variable. The HR for the entry time in years would be 1.785 (95% CI: [1.65,1.93]). We, therefore, concentrate on marginal quantities.

## 6 Simulation Study

### 6.1 Aims

The simulation study aims to demonstrate how estimates differ when the prerequisites of the analysis methods, such as external random censoring and the Markov assumption, are not fulfilled. We further investigate when a standard test used to check the Markov assumption detects a deviation from a Markov multistate model.

### 6.2 Data‐Generating Mechanism

We simulate 1000 datasets for each scenario and 600 patients, with 300 in each of the two treatment groups. First, the treatment group is randomly assigned, meaning that we simulate under the null of no treatment difference. The event times of the illness–death model are simulated as a nested series of competing risk settings (Beyersmann et al. 2011). The first event times are generated with the combined hazard $\lambda_{0.}\left(t\right)=\lambda_{01}\left(t\right)+\lambda_{02}\left(t\right)$ using the inversion method. Then, a binomial experiment decides with probability $\lambda_{01}\left(t\right)/\lambda_{01}\left(t\right)+\lambda_{02}\left(t\right)$ for the event of type 1, hospitalization, if an event occurs at t. For patients still at risk for an event, that is, those in state 1, the data‐generating mechanism is repeated, and the second event times are the first event times plus the waiting times simulated with the combined hazard $\lambda_{1.}\left(t\right)=\lambda_{10}\left(t\right)+\lambda_{12}\left(t\right)$, where time t is on the waiting timescale. The type of event 0, discharge from the hospital, has the conditional probability $\lambda_{10}\left(t\right)/\lambda_{10}\left(t\right)+\lambda_{12}\left(t\right)$. The algorithm is repeated until every patient is in the absorbing state 2. The progressive multistate model is simulated in the same way but using different transition hazards for each competing risk experiment. Random censoring is simulated according to the specified distribution, and each patient is censored if the censoring time happens before death. State‐dependent censoring is modeled as a third competing risk in each of the nested competing risk settings. In the Markovian multistate model, it acts as independent censoring because it does not change the other intensities (Aalen et al. 2008). The Markov property is violated as in Nießl et al. (2023) and explained below. In total, nine simulation settings are considered. Second, we consider simulations assuming a treatment difference that reduces the number of hospitalizations. Three hundred patients are simulated for each group with the algorithm as described. Random and state‐dependent censoring are generated as before, assuming that censoring does not depend on the treatment effect. The real data example inspires the simulation specifications. On average, between 210 and 245 patients are censored, and the mean maximum number of rehospitalizations per simulated dataset ranges from 8 to 12. Under the null of no treatment difference, the simulation specifications are as follows:

The first simulation setting is a Markovian illness–death model with recovery and random censoring. Corresponding to Figure 1, the transition hazards are $\lambda_{01}\left(t\right)=0.002,\;\lambda_{02}\left(t\right)=0.0007,\;\lambda_{10}\left(t\right)=0.004,\;\text{and}\;\lambda_{12}\left(t\right)=0.00075$. The censoring time is an exponentially distributed random variable with a rate of 0.00045.

The second simulation setting is a non‐Markov illness–death model with random censoring. The transition hazards and censoring process remain the same as before. However, by multiplying the event times by a gamma‐distributed frailty variable L∼Γ(2,1), the Markov assumption is violated. This means the event times are generated with individual transition hazards $\lambda_{i,hj}\left(t\right)=l_{i}\cdot \lambda_{hj}\left(t\right)$, given $L=l_{i}$ for individual i.

The third simulation setting is a Markovian illness–death model with state‐dependent censoring. The transition hazards are as before. The censoring time is modeled as a competing risk out of each nonterminating state with hazard $\lambda_{cens}\left(t\right)=0.00045\cdot \left(1+0.05\cdot \text{number previous states}\right)$. The cause‐specific censoring hazard increases with the number of previous states.

The fourth simulation setting is a non‐Markov illness–death model with state‐dependent censoring. The hazards and the state‐dependent censoring are chosen as before. The Markov assumption is violated as in the second simulation scenario.

The following four simulation scenarios are as in the first four scenarios. However, we now simulate a progressive model where the transition hazards of the illness–death model are multiplied by (1+0.1·number previous states). We simulate until an absorbing event (censoring/death) is reached and do not determine the maximum number of states.

Furthermore, we consider a ninth scenario with state‐entry‐time dependent transition hazards. The progressive model is simulated like in the sixth (second) scenario without a frailty variable but with hazards $\lambda_{01}\left(t\right)=0.002\cdot \left(\text{entry time into state 0}/1000\right)\cdot \left(1+0.1\cdot \text{number previous states}\right)$, $\lambda_{02}\left(t\right)=0.0007\cdot \left(\text{entry time into}\text{state 0}/1000\right)\cdot \left(1+0.1\cdot \text{number previous states}\right)$, $\lambda_{10}\left(t\right)=0.004\cdot \left(\text{entry time into state 1}/1000\right)\cdot \left(1\;+\;0.1\;\cdot \;\text{number previous states}\right)$, and $\lambda_{12}\left(t\right)=0.0011\cdot \left(\text{entry time into state 1}/1000\right)\cdot \left(1+0.1\cdot \text{number previous states}\right)$.

For the alternative, we reduced the hospitalization hazard for the treatment group $\lambda_{01}\left(t\right)=0.002\cdot \exp\left(-0.1625\right)$, resulting in an HR of 0.85.

R version 4.3.2 R Core Team (2024) was used for simulating the data. R uses the Mersenne twister for random number generation.

### 6.3 Estimand

The methods used for analyzing the simulated data each produce hazard ratios and a *p*‐value for the null hypothesis of no difference between treatment groups, no influence of the number of previous hospitalizations, and entry time. We mainly use the *p*‐value to evaluate the performance of the methods since it is the easiest to assess.

### 6.4 Methods for Analysis

We compute an AG model for the illness–death models and a PWP model for the progressive multistate models. Each model is estimated twice, one time with covariable treatment and the covariable testing the Markov assumption (previous hospitalizations or entry time; model 1) and one time only with covariable treatment (model 2). The cumulative hazard/rate, the average length of stay, the state occupation probability, and the expected number of recurrent events are investigated under different censoring and Markov patterns. The R packages survival, etm, and mets were used for analysis. R‐code is available in the Supporting Information.

### 6.5 Performance Measure

We count the number of rejections of the Wald test statistic with the null hypothesis of no influence, both for the covariable measuring the treatment difference and the covariable testing the Markov property, at a significance level of 5%. Since we simulate the data under the null for the two treatment groups, meaning no treatment difference, we expect the number of rejections of a significant influence of the treatment variable to be approximately 50 in 1000 simulation runs at a significance level of 5%. The Markov test, which assesses the significance of the number of previous hospitalizations (Scenarios 1–4) and the entry time (Scenarios 5–9), should detect an influence in the non‐Markov scenarios 2, 3, 6, 8, and 9. Additionally, the Nelson–Aalen estimator is used to illustrate the cumulative hazards and rates, and the Aalen–Johansen estimator for the average length of stay, state occupation probability, and expected number of recurrent events in the different underlying censoring schemes.

### 6.6 Results of the Simulation

The results of the simulation study under the null hypothesis are shown in Table 2. We observe that the covariable previous hospitalization has a significant influence on the non‐Markov illness–death models as the number of rejections increases substantially. However, if we include only the treatment variable, we see a similar number of rejections of the null hypothesis of no treatment difference in Markov and non‐Markov models, although the Andersen–Gill model requires the Markov assumption. Furthermore, the censoring scheme does not influence the rejection of the null hypothesis, and we obtain comparable results for the illness–death model with both random and state‐dependent censoring. For testing the Markov assumption in the progressive multistate model, we include the entry time into the state “at home.” This test shows that, in our settings, there is no deviation from the Markov property, besides the probability of error. The covariable entry time shows a significant influence in the ninth scenario, with entry time dependent transition hazards. The simulations in Table 2 show that one can test the Markov assumption using a semiparametric model, but this does not guarantee the detection of all deviations from the Markov property. The results for the Markov tests are comparable under the alternative of a treatment effect. The number of rejected hypothesis of “no treatment effect” is between 200 and 570 and higher in the Markovian than in the non‐Markovian models. The power is too small. The results are presented in Table S1.

**TABLE 2:** Results for the simulation settings under the null of no treatment effect: Number of rejected hypothesis of “no effect of the covariable” with a significance level 5%. IDM: illness–death model, MSM: progressive multistate model, rand.cens: random censoring, st.dep.cens: state‐dependent censoring, AG: Andersen–Gill model, PWP: Prentice–Williams–Peterson model, and prev. hosp: previous hospitalizations. The ninth scenario is marked with (*). The Markov property is destroyed by entry time‐dependent hazards and not with a frailty variable as in the eighth scenario. The boxes highlight the results where 950 rejections of the Markov test were expected but not observed.

	**Model 1**			**Model 2**
	**Rejected tests of 1000 simulations**			
**Simulation setting**	**Model**	**Prev. hosp**.	**Treatment**	**Treatment**
1. Markov, IDM, rand.cens.	AG	57	41	44
2. non‐Markov, IDM, rand.cens.	AG	1000	50	58
3. Markov, IDM, st.dep.cens.	AG	61	42	40
4. **non‐Markov, IDM, st.dep.cens**.	AG	1000	47	55
	**Model**	**Entry time**	**Treatment**	**Treatment**
5. Markov, MSM, rand.cens.	PWP	58	65	66
6. non‐Markov, MSM, rand.cens.	PWP	86	61	64
7. Markov, MSM, st.dep.cens.	PWP	77	57	59
8. **non‐Markov, MSM, st.dep.cens**.	PWP	94	60	62
9. **non‐Markov, MSM, st.dep.cens**.(*)	PWP	1000	56	77

**FIGURE 9:**
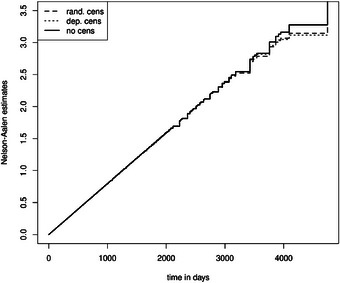
Cumulative transition hazard of transition 0 to 1 in a Markov illness–death model.

**FIGURE 10:**
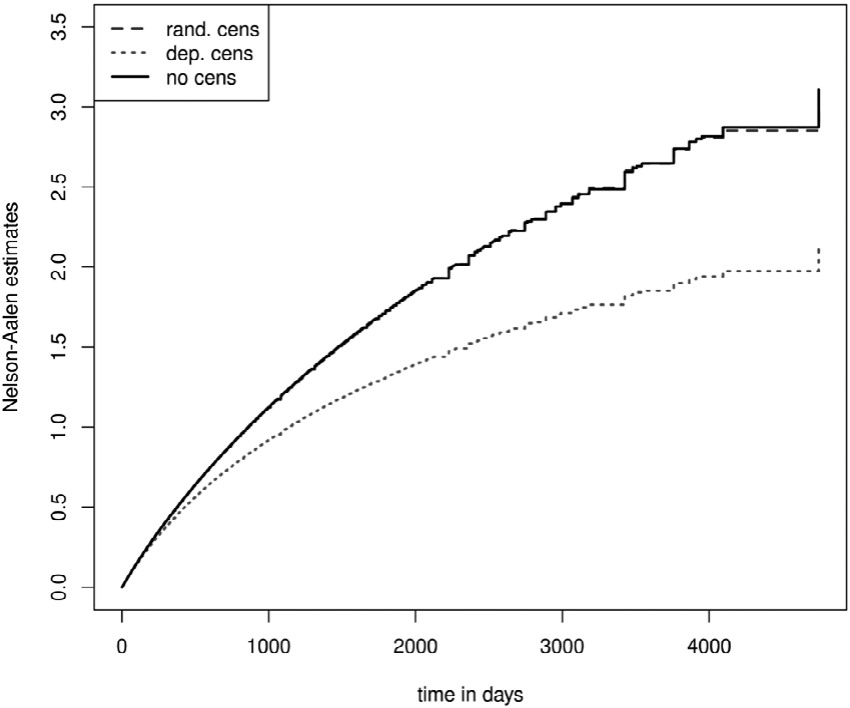
Cumulative partly conditional transition rate of transition 0 to 1 in a non‐Markov illness–death model.

In addition, we aim to demonstrate that the prerequisite random censoring is essential for estimating partly conditional transition rates, the average length of stay, the state occupation probability, and the expected number of recurrent events. Furthermore, we show that partly conditional transition rates in non‐Markov models are not the same as the hazard in Markov models (Furberg et al. 2022). Figure 9 shows the mean Nelson–Aalen estimator of 1000 simulated datasets in a Markov illness–death model (dashed: Scenario 1, dotted: Scenario 3, black: without censoring). We see that random and independent censoring do not, on average, disturb the estimation of the cumulative hazard, save for late time points. This implies that the Nelson–Aalen estimator is a consistent estimator in settings with both random and independent censoring. Figure 10 shows the mean Nelson–Aalen estimator of 1000 simulated datasets in a non‐Markov illness–death model (dashed: Scenario 2, dotted: Scenario 4, black: without censoring). We have used frailties to violate the Markov property, which results in a non‐constant and a smaller cumulative rate compared to the cumulative hazard in Figure 9 (Aalen et al. 2008). Most importantly, we see a pronounced bias when we estimate the partly conditional transition rate when state‐dependent censoring is present: The estimated partly conditional transition rate is represented by the dotted curve and is below the ones in the settings with random and no censoring. State‐dependent censoring, which is independent but not random censoring, destroys the i.i.d. assumption of the multistate process, which is necessary for consistent rate estimation in non‐Markov models (Nießl et al. 2023).

The mean of the average length of stay, the state occupation probability in state 2 (1 − overall survival probability), and the expected number of recurrent events are presented in Figures S9– S14. These figures show that the estimates are biased in the non‐Markov models with state‐dependent censoring.

## 7 Discussion

The analysis of multiple events per individual in clinical studies remains a challenging task. We aimed to provide a step‐by‐step guidance for the applied researcher on how common time‐to‐first event analysis can be extended to multiple time‐to‐event analysis, giving special attention to the underlying model assumptions. Methods for recurrent time‐to‐events with a competing terminating event, based on the Cox proportional hazards model, were considered, as well as multistate approaches. A simulation study showed the importance of considering model assumptions. A real data example was used for illustration.

We emphasized that intensity‐based models require a full specification of the recurrent event process, that is, there is a risk of model miss‐specification. Nevertheless, the intensity models, as well as the (partly conditional) rate approaches (non‐ and semiparametric like the Ghosh and Lin model) were essentially in practical agreement. Estimating probabilities completes the understanding of the treatment effect. Only when estimating transition probabilities conditioning on a time greater than 0 there exists a model misspecification in the absence of the Markov property. An extended multistate model with less restrictive model assumptions (Model 2) requires a sufficiently large number of patients at risk and with an observed transition. Therefore, the estimation of transition probabilities and hazards often provides too wide confidence intervals for a proper treatment comparison, as in our case study. Easily interpretable marginal quantities like the mean number of hospitalizations, the average length of stay in hospital, and the state occupation probabilities can still be consistently estimated. Kim et al. (2021) promote the usage of marginal features in clinical trials and their causal interpretation. However, the semiparametric models also allow the inclusion of other covariables. For example, a model accounting for age, sex, and the New York Heart Association functional class may describe the disease and the treatment effect more precisely. Including other covariables can also relax the dependence on previous events, which was our test for the Markov property. Some treatment effects which are measured in the hazard ratio can be hidden in the stratification in the PWP model (Furberg et al. 2022; Kim et al. 2021). A possible extension of the model is the inclusion of strata‐specific effects, that is, transition‐specific effects (Kim et al. 2021). However, one is often interested in describing the entire event process within a single model (i.e., all‐cause hazard) and estimating a single treatment effect for all events (hospitalization and death). Hence, a composite endpoint would be of interest. Ozga et al. (2018) showed how the so‐called AG model and the PWP model can be used in the setting of a composite endpoint where it is possible to have another event immediately after experiencing a nonfatal event (e.g., the recurrent event of a heart attack and the absorbing event of death). We saw in our data example that the results from the nonparametric estimators complement the semiparametric results. The time‐to‐first event analysis indicates that treatment has no detrimental effect on survival. It works to reduce first hospitalizations. Considering also recurrent events, we observed that admission to and discharge from the hospital are very common. The treatment for this disease works by reducing the average length of hospital stay, the mean number of hospitalizations, and slightly extending overall survival.

In our work, we focused on the methods that are easy to use. We emphasized the use of marginal quantities like the mean number of hospitalizations and the average length of stay, which are not yet commonly used in practice. Especially explaining these quantities with the multistate models perspective helps the applied researcher to interpret a treatment effect. However, other strategies and models exist for analyzing recurrent events. Ma et al. (2023) propose a multistate model framework and identify covariables that influence the hazard in both time‐constant and time‐varying manner. Furberg et al. (2023) estimate marginal models with the help of pseudo‐observations. Erdmann et al. (2024) compare different nonparametric estimators with varying underlying censoring schemes for the marginal expected number of recurrent events also in the progressive multistate model, whereas Cortese and Scheike (2022) investigate and improve the efficacy of one nonparametric estimator with underlying random censoring. Furthermore, Bouaziz (2024) evaluates the prediction ability of different models for the expected number of recurrent events using a new score. Bühler et al. (2022) compare the Cox model with two marginal rate‐based methods (the negative binomial and the Lin–Wei–Yang–Ying model for recurrent event processes without a terminal event). Hougaard (2022) considers the timescale for the analysis of recurrent events and shows disadvantages of a gap‐time approach. Titman and Putter (2022) develop a new test for the Markov property under random censoring and compare it to the test used in this study. An overview of Markov tests can also be found in Soutinho and Meira‐Machado (2022).

We did not consider those topics because we aimed to focus on existing methods and give a step‐wise analysis guideline, where we emphasized the importance of considering model assumptions. Furthermore, we advertise the multistate model thinking, as it helps to clarify what is estimated and can also aid in formalizing the estimand. We recommend a step‐wise analysis procedure, extending the time‐to‐first event analysis to more complex models and using the not‐yet‐common state occupation probabilities, the mean number of recurrent events, and the average length of stay for a causal interpretation in a randomized trial. To evaluate a treatment difference, there is no one and only estimator. We conclude that a mixture of several analyses can provide a more complete picture of the treatment differences, which echoes a recommendation by Lin (1994). If, in a randomized trial, the decision is to consider a recurrent endpoint as primary, we consider an analysis based on partly conditional transition rates as a natural choice. However, more research is needed on how event‐driven censoring may potentially impact such an analysis.

The multistate approach used in the present paper is quite generic. In the context of our application, one assumes that there may be multiple hospitalizations and discharges from the hospital over the course of time. Death is naturally modeled as a “competing” absorbing state, but somewhat metaphysical constructs such as hospitalization after death are not considered. Two reviewers suggested semicompeting risks or joint models as alternative approaches. The former is an illness–death model, however, with the potential aim of investigating, say, time‐to‐hospitalization, possibly after death, as an outcome. (See, e.g., Andersen and Keiding 2012 for a critique.) Joint modeling mostly focuses on two processes that run in parallel, one for time‐to‐event and one for a time‐dependent marker, the latter typically continuous. The processes are, for example, linked by a shared frailty, and there are suggestions for categorical markers, too (Choi et al. 2015).

## Conflicts of Interest

The authors declare no conflicts of interest.

## Ethics Statement

Approval was obtained from all responsible ethics committees of the study centers. All corresponding protocol amendments for the increased sample size and longer term follow‐up received ethics approval, and trial registration details were updated (ISRCTN23325295).

## Open Research Badges

This article has earned an Open Data badge for making publicly available the digitally‐shareable data necessary to reproduce the reported results. The data is available in the Supporting Information section.

This article has earned an open data badge “**Reproducible Research**” for making publicly available the code necessary to reproduce the reported results. “The results reported in this article could fully be reproduced.”

## Supporting information


**Supporting File 1:** bimj70107‐sup‐0002‐Datacode.zip.

**Supporting File 2:** bimj70107‐sup‐0001‐SuppMat.pdf.

## Data Availability

The data that support the findings of this study are available from the senior author, AO, upon reasonable request. The R‐code, including the simulation study, is available in the Supporting Information.
