# Supplementary material for: The Challenge of Time‐to‐Event Analysis for Multiple Events: A Guided Tour From Time‐to‐First‐Event to Recurrent Time‐to‐Event Analysis
Source: Biom J. 2026 Jan 28;68(1):e70107. doi: 10.1002/bimj.70107 (PMC12848661; doi:10.1002/bimj.70107)
Supplement: Supplementary file 1 — Supporting File 1: bimj70107‐sup‐0002‐Datacode.zip. [file BIMJ-68-e70107-s001.pdf]

Biometrical Journal **52** (2010) 61, zzz–zzz / DOI: 10.1002/bimj.200100000

9/12/2025

Appendix to: The challenge of time-to-event analysis for multiple events: a guided tour from time to-first-event to recurrent time-to-event analysis

Sandra Schmeller[*,1], **Alexandra Erdmann** [1], **Jan Beyersmann** [1], **Christiane Angermann** [2], and **Ann-Kathrin Ozga** [3]

[1] Institute of Statistics, Ulm University, Ulm, Germany
[2] Comprehensive Heart Failure Centre, University and University Hospital Würzburg, Würzburg, Germany, and Department of Internal Medicine I, University Hospital Würzburg, Würzburg, Germany
[3] Institute of Medical Biometry and Epidemiology, University Medical Center Hamburg-Eppendorf, Hamburg, Germany

Received zzz, revised zzz, accepted zzz

Key words: Method-comparison; Multi-state model; Recurrent events; Markov assumption

Section 1 shows figures from the E-INH-trial shown in the main manuscript but with 95%-confidence interval as well as figures which are not shown in the main manuscript for reasons of space. Section 2 demonstrates results from the simulation study under the alternative of a treatment effect. Plots for the average length of stay, expected number of hospitalizations and the state occupation probability in the Markov and non Markov illness death model with different censoring patterns are shown.

1 Real data application

*Corresponding author: e-mail: sandra.schmeller@uni-ulm.de, Phone: +49(0)731/50-33102

© 2010 WILEY-VCH Verlag GmbH & Co. KGaA, Weinheim

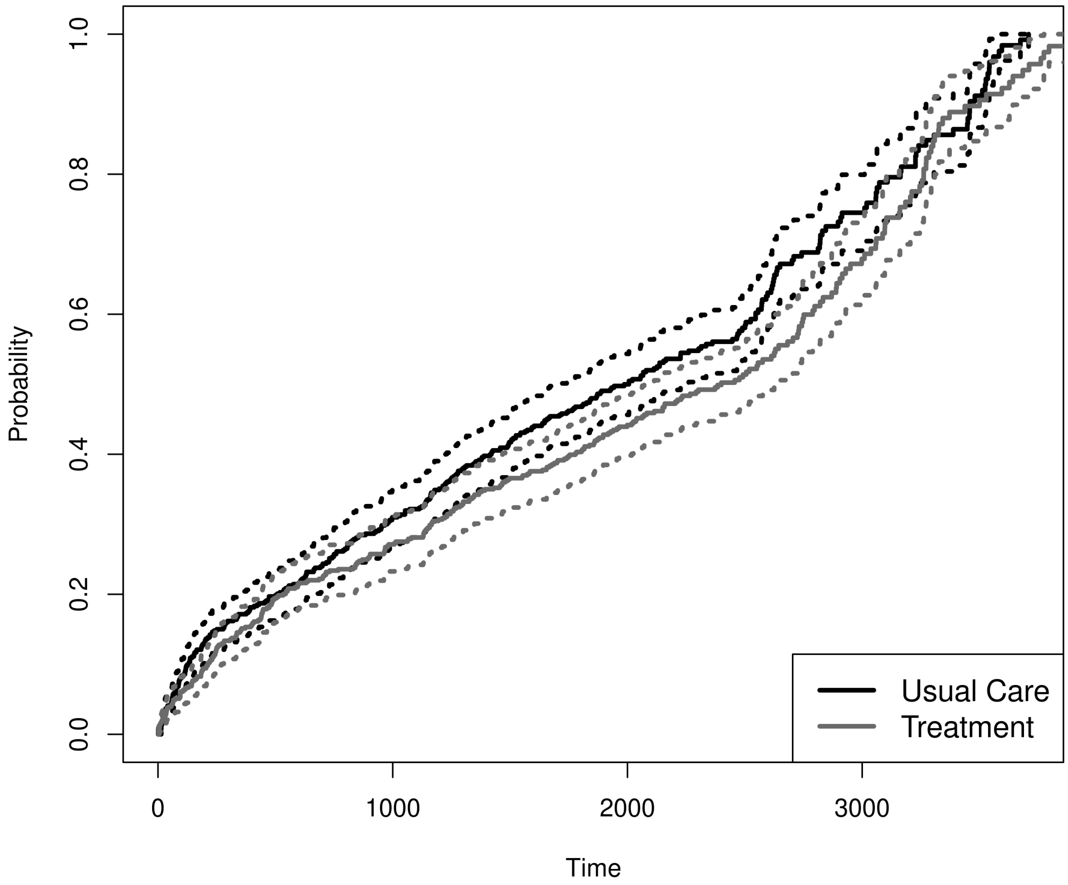

Figure 1 Kaplan-Meier estimates for one minus the overall survival probability with 95%− confidence interval (dashed line).

© 2010 WILEY-VCH Verlag GmbH & Co. KGaA, Weinheim

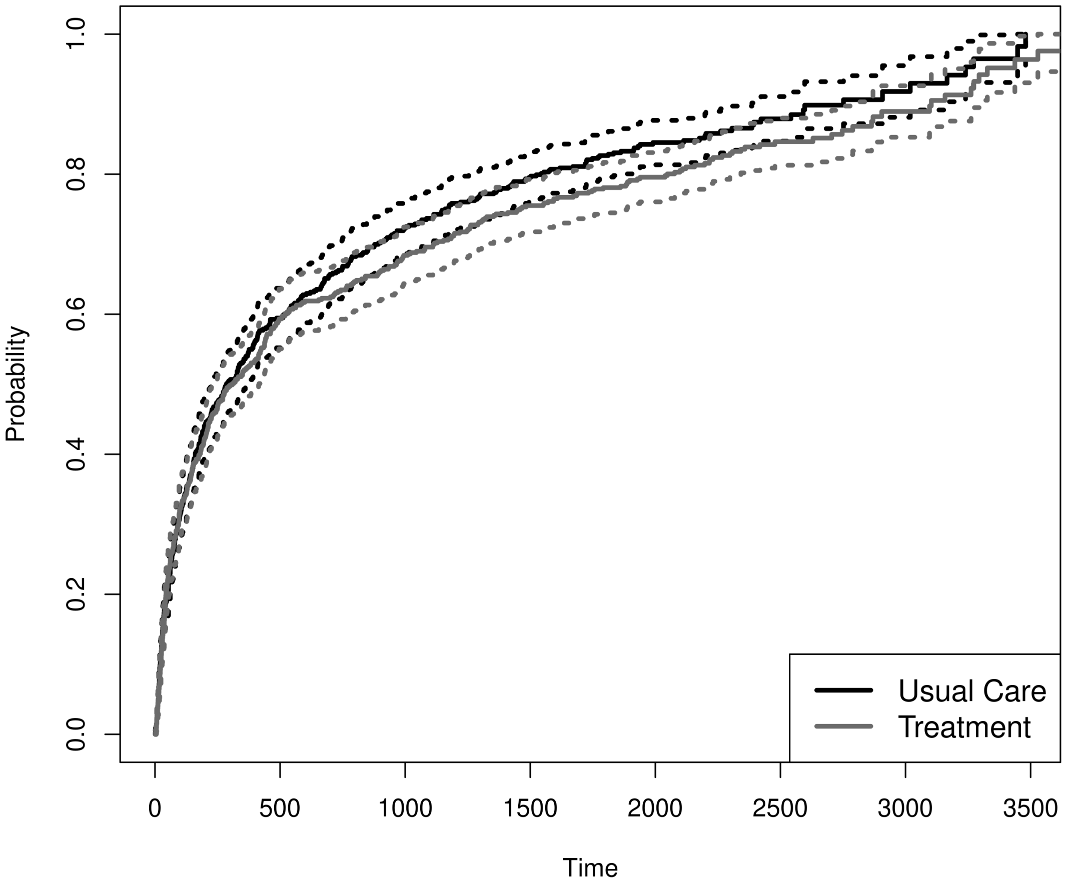

Figure 2 Kaplan-Meier estimates for one minus the hospital-free survival probability with 95%− confidence interval (dashed line).

© 2010 WILEY-VCH Verlag GmbH & Co. KGaA, Weinheim

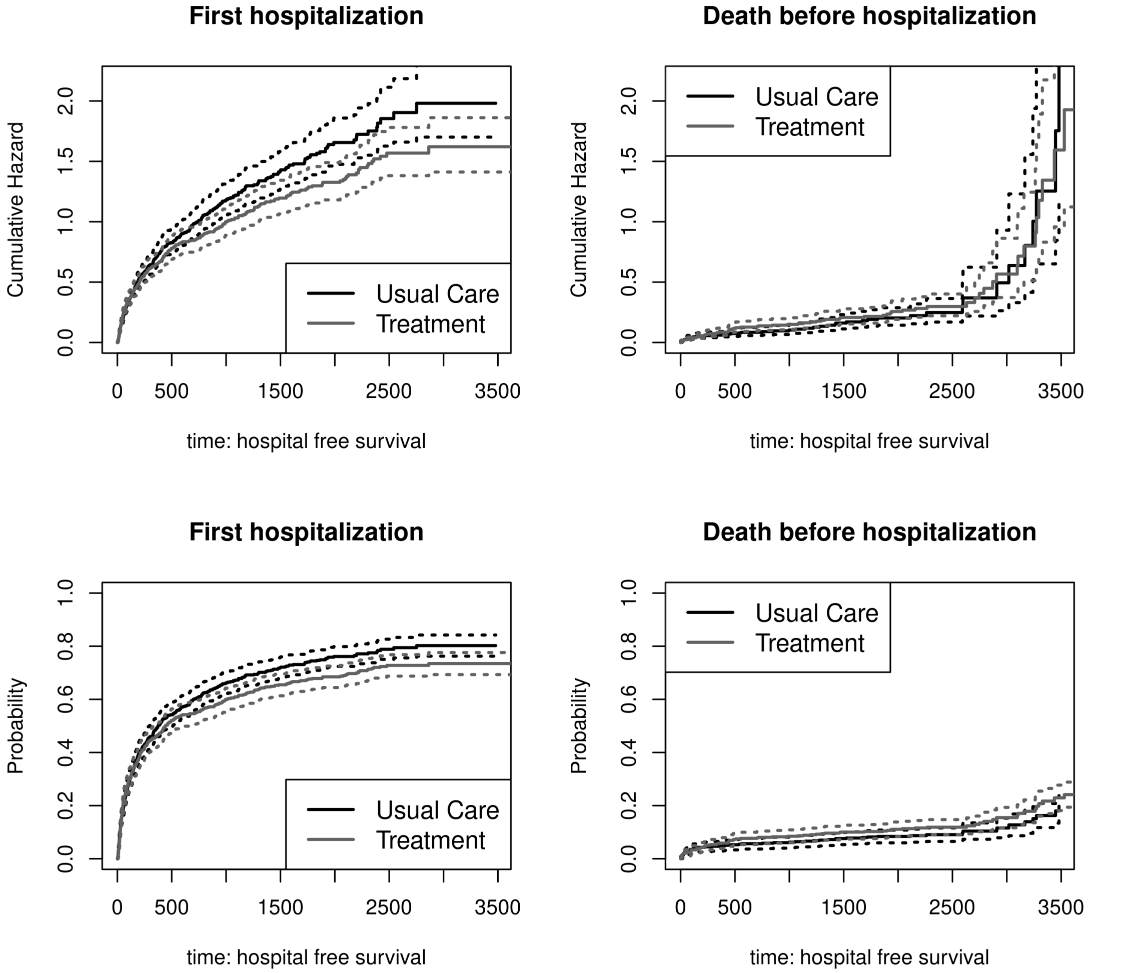

Figure 3 Nelson-Aalen (first row) and Aalen-Johansen (second row) estimates for the competing risks first hospitalization and death before hospitalization with 95%− confidence interval (dashed line).

© 2010 WILEY-VCH Verlag GmbH & Co. KGaA, Weinheim

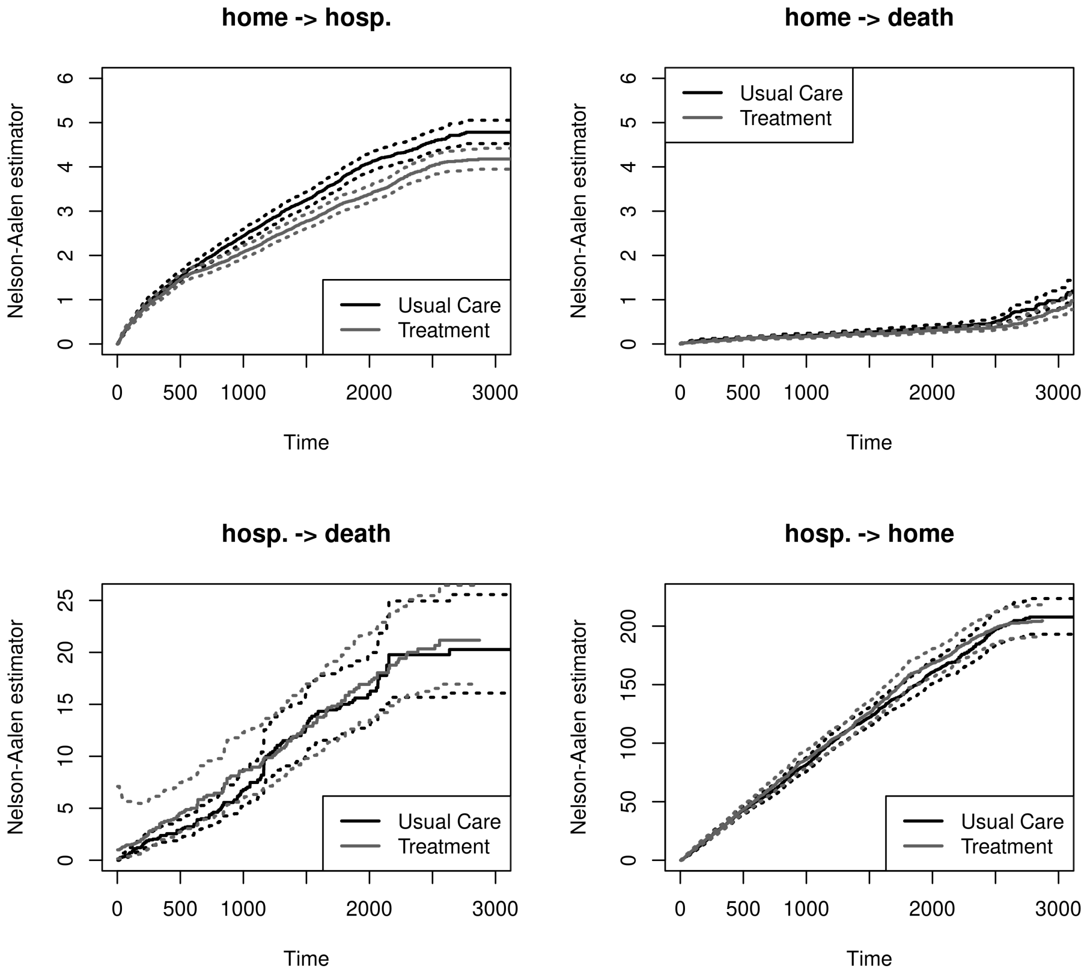

Figure 4 Nelson-Aalen estimates in the illness-death model with 95%− confidence interval (dashed line). hosp.: hospitalization

© 2010 WILEY-VCH Verlag GmbH & Co. KGaA, Weinheim

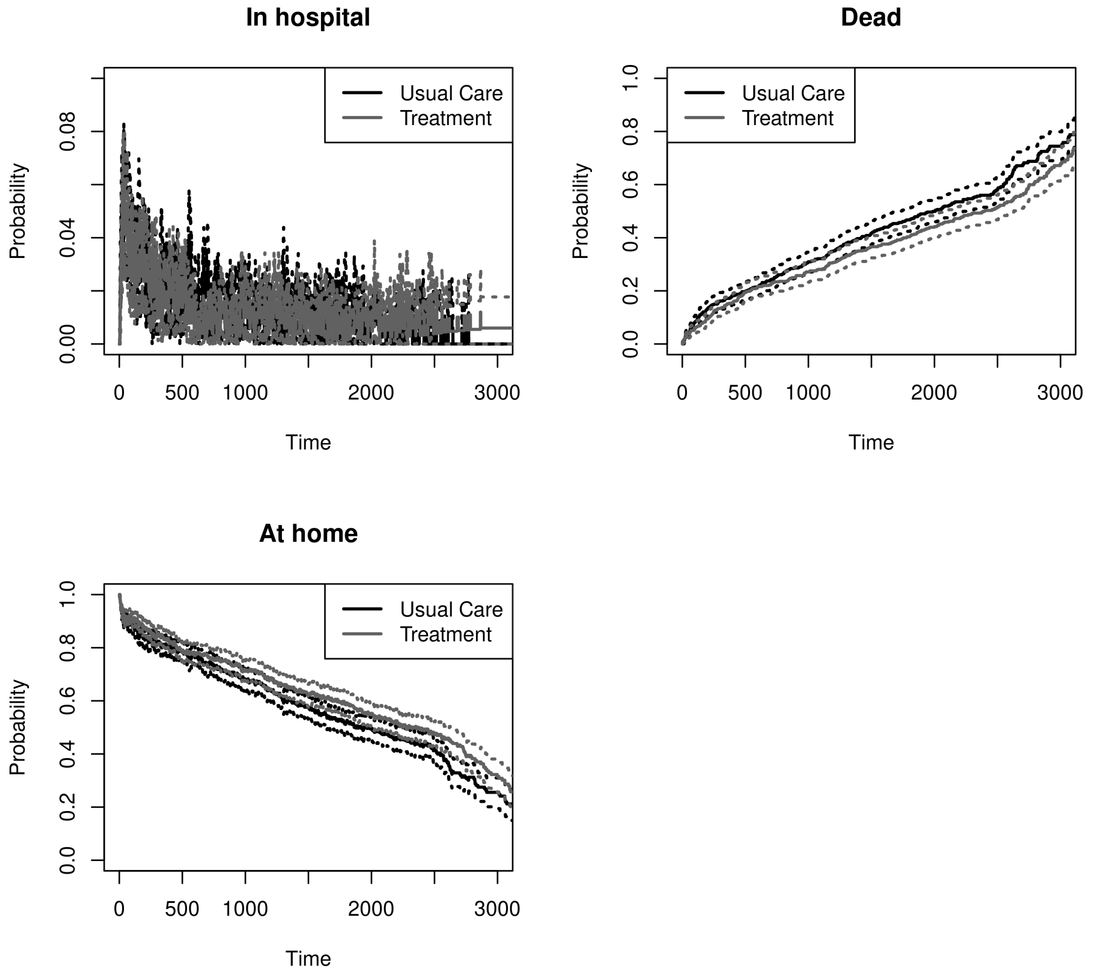

Figure 5 State occupation probabilities (Aalen-Johansen estimates) in the illness-death model with 95%− confidence interval (dashed line).

© 2010 WILEY-VCH Verlag GmbH & Co. KGaA, Weinheim

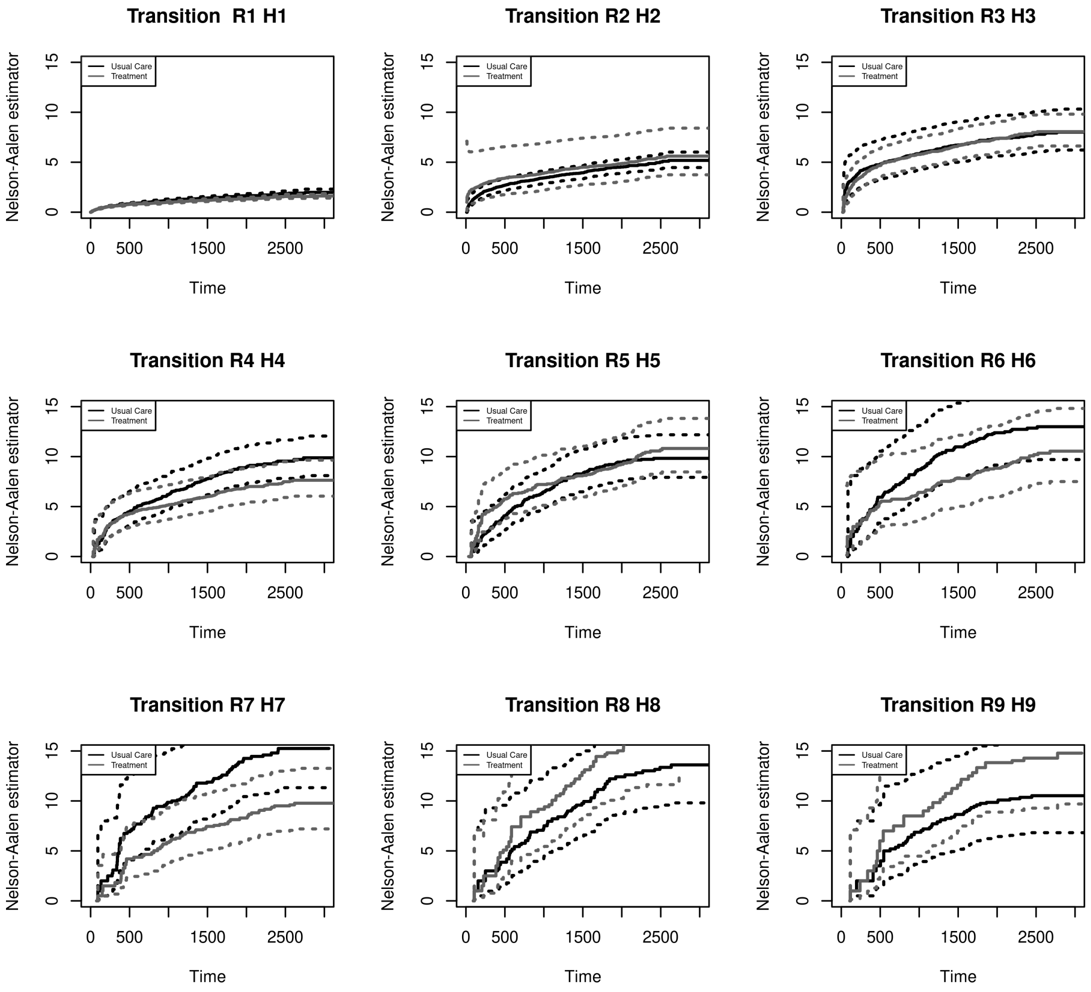

Figure 6 Nelson-Aalen estimates with 95%− confidence interval (dashed line) in the progressive multistate model. Transitions: At home (R) to Hospitalization (H)

© 2010 WILEY-VCH Verlag GmbH & Co. KGaA, Weinheim

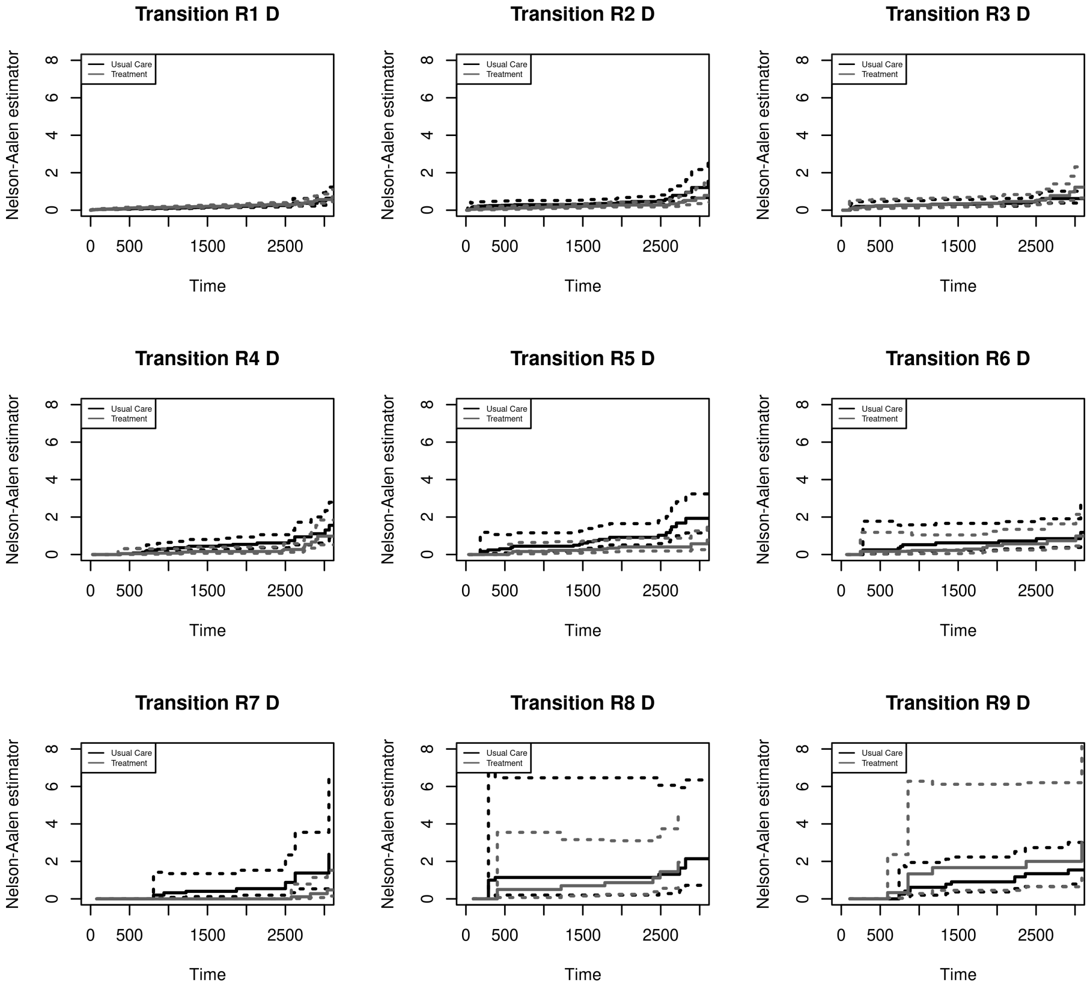

Figure 7 Nelson-Aalen estimates with 95%− confidence interval (dashed line) in the progressive multistate model. Transitions: At home (R) to Death (D)

© 2010 WILEY-VCH Verlag GmbH & Co. KGaA, Weinheim

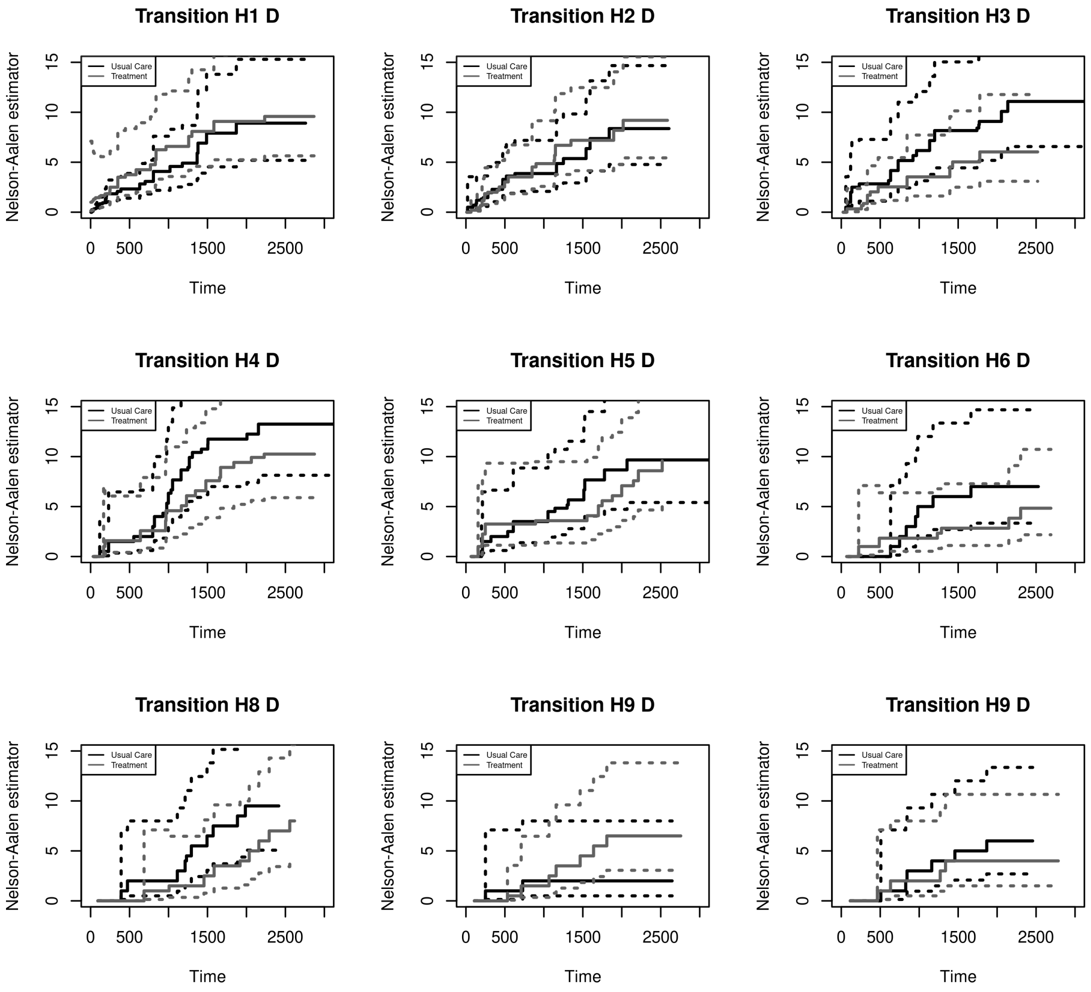

Figure 8 Nelson-Aalen estimates with 95%− confidence interval (dashed line) in the progressive multistate model. Transitions: Hospital (H) to Death (D)

© 2010 WILEY-VCH Verlag GmbH & Co. KGaA, Weinheim

2 Simulation study

	**model 1**			**model 2**
**Simulation setting**	**rejected tests of 1000 simulations**			
	**model**	**prev. hosp.**	**treatment**	**treatment**
1. Markov, IDM, rand.cens.	AG	63	572	577
2. non Markov, IDM, rand.cens.	AG	1000	378	352
3. Markov, IDM, st.dep.cens.	AG	61	534	538
4. **non Markov, IDM, st.dep.cens.**	AG	1000	351	331
	**model**	**entry-time**	**treatment**	**treatment**
5. Markov, MSM, rand.cens.	PWP	68	322	318
6. non Markov, MSM, rand.cens.	PWP	73	198	197
7. Markov, MSM, st.dep.cens.	PWP	73	309	306
8. **non Markov, MSM, st.dep.cens.**	PWP	95	193	195
9. **non Markov, MSM, st.dep.cens.(∗)**	PWP	1000	252	216

Table 1 Results for the simulation settings under the alternative of a treatment effect: Number of rejected hypothesis of 'no effect of the covariable' with a significance level 5%. IDM: illness-death model, MSM: progressive multistate model, rand.cens: random censoring, st.dep.cens: state -dependent censoring, AG: Andersen-Gill model, PWP: Prentice Williams Peterson model, prev. hosp: previous hospitalizations. The ninth scenario is marked with (∗). The Markov property is destroyed by entry-time dependent hazards and not with a frailty variable as in the eighth scenario. The boxes highlight the results where 950 rejections of the Markov test were expected but not observed.

© 2010 WILEY-VCH Verlag GmbH & Co. KGaA, Weinheim

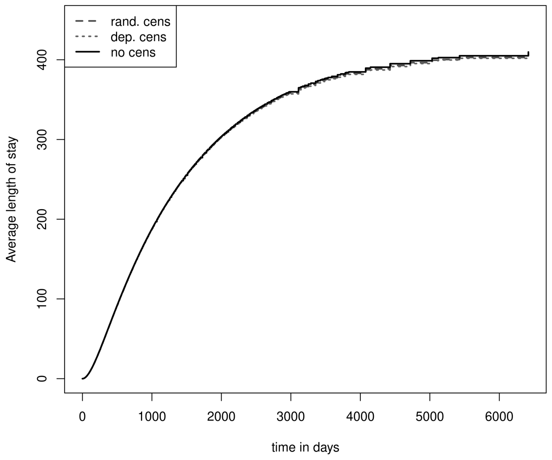

Figure 9 Average length of stay in state 1 of the Markovian illness-death model.

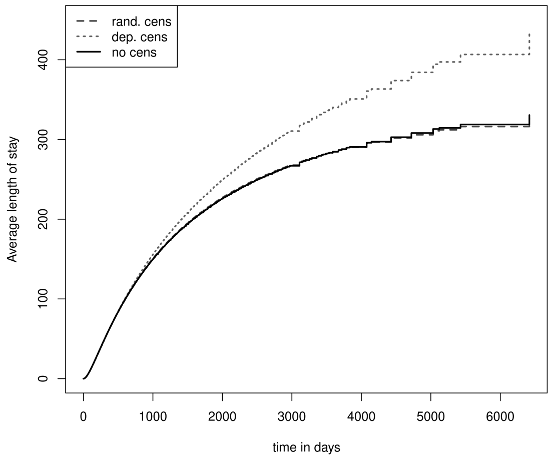

Figure 10 (Biased) Average length of stay in state 1 of the non Markovian illness-death model.

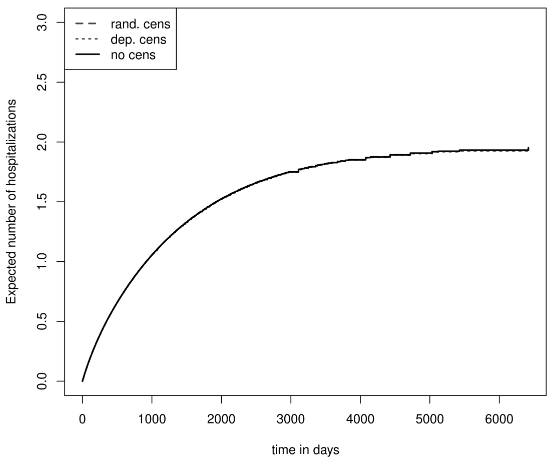

Figure 11 Expected number of recurrent events in the Markovian illness-death model.

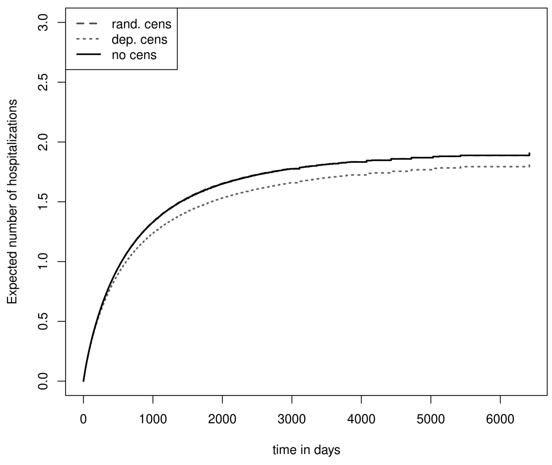

Figure 12 (Biased) Expected number of recurrent events in the non Markovian illness-death model.

© 2010 WILEY-VCH Verlag GmbH & Co. KGaA, Weinheim

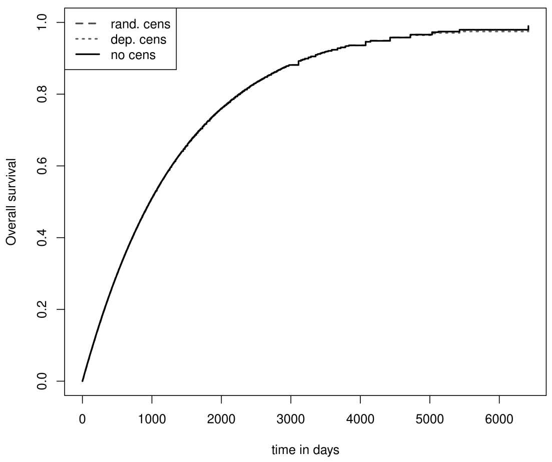

Figure 13 1- Overall survival probability (state occupation probability in state 2) in the Markovian illness-death model.

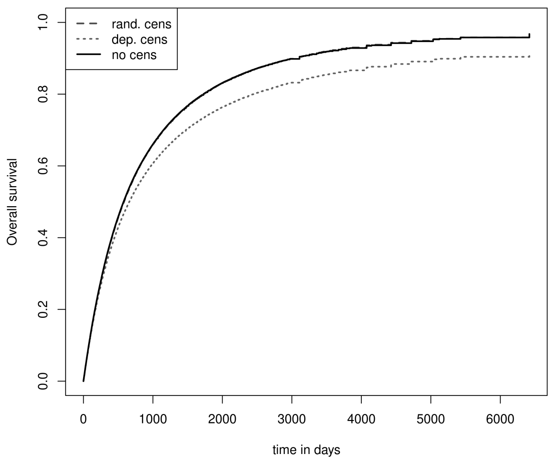

Figure 14 (Biased) 1- Overall survival probability (state occupation probability in state 2) in the non Markovian illness-death model.

© 2010 WILEY-VCH Verlag GmbH & Co. KGaA, Weinheim
