# Supplementary material for: The Challenge of Time‐to‐Event Analysis for Multiple Events: A Guided Tour From Time‐to‐First‐Event to Recurrent Time‐to‐Event Analysis
Source: Biom J. 2026 Jan 28;68(1):e70107. doi: 10.1002/bimj.70107 (PMC12848661; doi:10.1002/bimj.70107)
Supplement: Supplementary file 2 — Supporting File 2: bimj70107‐sup‐0001‐SuppMat.pdf. [file BIMJ-68-e70107-s002.zip › R_Files/readme.pdf]

README

Code supplement for the manuscript "The challenge of time-to-event analysis for multiple events: a guided tour from time to-first-event to recurrent time-to-event analysis"
by S. Schmeller, A. Erdmann, J. Beyersmann, C. Angermann, A.K. Ozga

For questions, comments or remarks about the code please contact S. Schmeller (sandra.schmeller@uni-ulm.de)

The code has been written using R version 4.4.1 (platform x86_64-pc-linux-gnu) with packages parallel, stats, graphics, grDevices, utils, datasets, methods, base, mets_1.3.4, timereg_2.0.5, reReg_1.4.6, latex2exp_0.9.6, lattice_0.22-5, mvna_2.0.1, dplyr_1.1.4, survival_3.7-0, etm_1.1.1, haven_2.5.4.

Results and intermediate results from the data example are saved in the folder "Results_Study" and from the simulations in the folder "Results_Simulation".

Application_t_E_INHStudy.R : R-code for Section 5, main document and Section 1, Appendix. We provide a simulated dataset that mimics the main features of the real data analyzed in the article. The bootstrap repetition for the expected number of hospitalizations was done on the HPC-BW cluster because it is computational expensive (Code for 5 iterations is included). Interim results are saved in meanBTherapydataDummy.RData and meanBControldataDummy.RData in the folder Results_Study that everything else can be compiled overnight.
The code requires the mets package version 1.3.4 (an updated version does not work). Please specify the paths in line 19 and 25.

Simulations (Section 6, main document):

R-Files that don't need to be run (because they are called from other files):
- *Fkt_sim_IDMRandomCens.R* : Function to simulate data with random censoring; both for the Illness death model with recovery and the progressive multistate model (for Scenario 1, 5, 2, 6)
- *Fkt_sim_IDMDepCens.R* : Function to simulate data with state dependent censoring both for the Illness death model with recovery and the progressive multistate model (for Scenario 3, 7, 4, 8)
- *Fkt_sim_IDMNonMarkovEntrydep.R* : Function to simulate data for a non Markov model (with dependency on the entry time) for the progressive multistate model (Scenario 9)

Run these R-files first: The following R-Files need to be compiled to get the results for table 2, main manuscript. Furthermore, datasets are simulated which are also used in Simulation_plots.R. The code contains mclapply for a parallel computing. This function requires a linux system.
- *simulation_RandomCens.R* : Simulates the data and calculates the results presented in table 2, main manuscript (for scenarios 1, 5, 2, 6). Calls Fkt_sim_IDMRandomCens.R to simulate the data. Please specify the paths in line 9 and 12. In line 27 the number of cores need to be specified.
- *simulation_DepCens.R* : Simulates the data and calculates the results presented in table 2, main manuscript (for scenarios 3, 7, 4, 8). Calls Fkt_sim_IDMDepCens.R to simulate the data. Please specify the paths in line 9 and 12. In line 27 the number of cores need to be specified.
- *simulation_NonMarkovEntrydep.R* : Simulates the data and calculates the results presented in table 2, main manuscript (for scenario 9). Calls Fkt_sim_IDMNonMarkovEntrydep.R to simulate the data. Please specify the paths in line 9 and 12. In line 93 the number of cores need to be specified.

The following R-Files need to be compiled to get the results for table 1, Appendix. The code contains mclapply for a parallel computing. This function requires a linux system.

- *H1_simulation_RandomCens.R* : Simulates the data and calculates the results presented in table 1, Appendix (for scenarios 1, 5, 2, 6). Calls Fkt_sim_IDMRandomCens.R to simulate the data. Please specify the paths in line 9 and 12. In line 27 the number of cores need to be specified.
- *H1_simulation_DepCens.R* : Simulates the data and calculates the results presented in table 1, Appendix (for scenarios 3, 7, 4, 8). Calls Fkt_sim_IDMDepCens.R to simulate the data. Please specify the paths in line 9 and 12. In line 27 the number of cores need to be specified.
- *H1_simulation_NonMarkovEntrydep.R* : Simulates the data and calculates the results presented in table 1, Appendix (for scenario 9). Calls Fkt_sim_IDMNonMarkovEntrydep.R to simulate the data. Please specify the paths in line 9 and 12. In line 27 the number of cores need to be specified.

Simulation_plots.R: (can be run after simulation_RandomCens.R, simulation_DepCens.R, simulation_NonMarkovEntrydep.R) Can be compiled within 48h. The code contains mclapply for a parallel computing. This function requires a linux system. Please specify the paths in line 21 and 24. In line 200 the number of cores need to be specified. The code requires the mets package version 1.3.4 (an updated version does not work).
Calculates the estimates of
-mean number of hospitalizations (Fig 11, 12 in Appendix)
-average length of stay (Fig 9, 10 in Appendix)
-Hazard and partly cond. transition rates (Fig. 9,10 in main document)
-state occupation probability in state 2 (Fig. 13, 14 in Appendix)
with the *previous* simulated IDM data (in simulation_RandomCens.R, simulation_DepCens.R, simulation_NonMarkovEntrydep.R). Furthermore, simulates data with no censoring Markovian and non Markovian (calls *Fkt_sim_IDMRandomCens.R).*
