# Supplementary figures and images for: The Challenge of Time‐to‐Event Analysis for Multiple Events: A Guided Tour From Time‐to‐First‐Event to Recurrent Time‐to‐Event Analysis
Source: Biom J. 2026 Jan 28;68(1):e70107. doi: 10.1002/bimj.70107 (PMC12848661; doi:10.1002/bimj.70107)

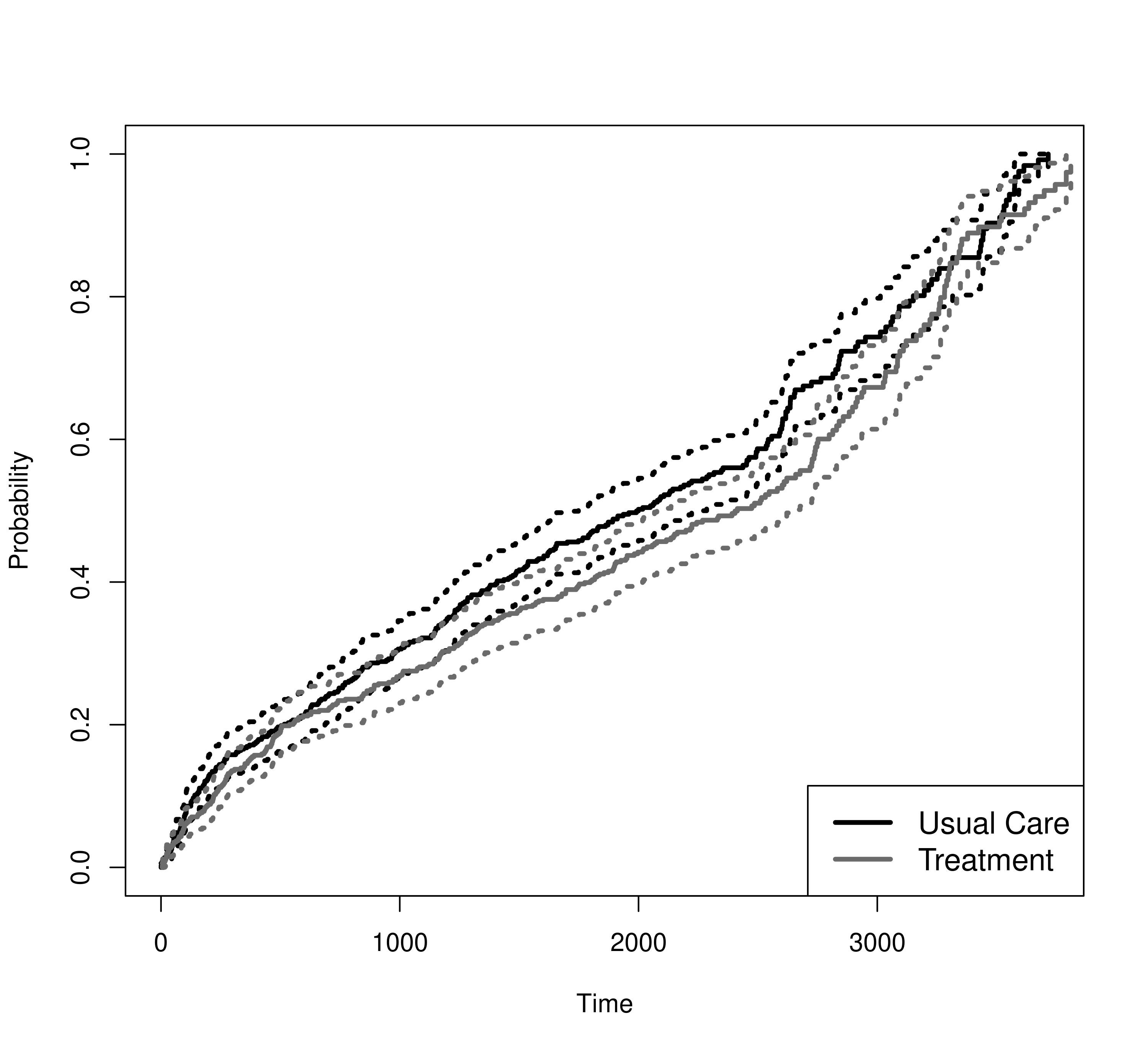

Supplement: Supplementary file 2 — Supporting File 2: bimj70107‐sup‐0001‐SuppMat.pdf. [file BIMJ-68-e70107-s002.zip › R_Files/Results_Study/Fig1_App_OSRev.png]

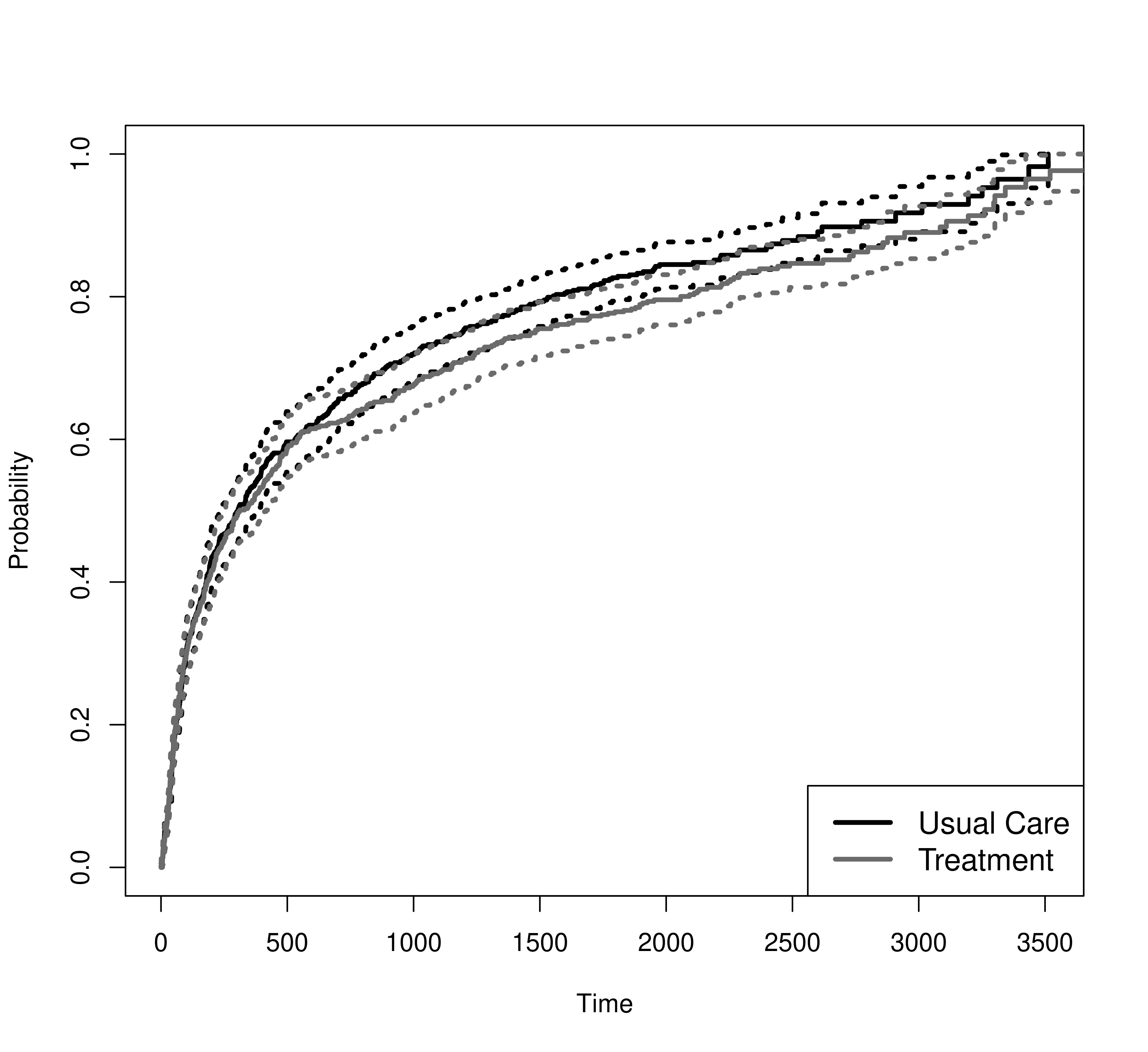

Supplement: Supplementary file 2 — Supporting File 2: bimj70107‐sup‐0001‐SuppMat.pdf. [file BIMJ-68-e70107-s002.zip › R_Files/Results_Study/Fig2_App_HFSRev.png]

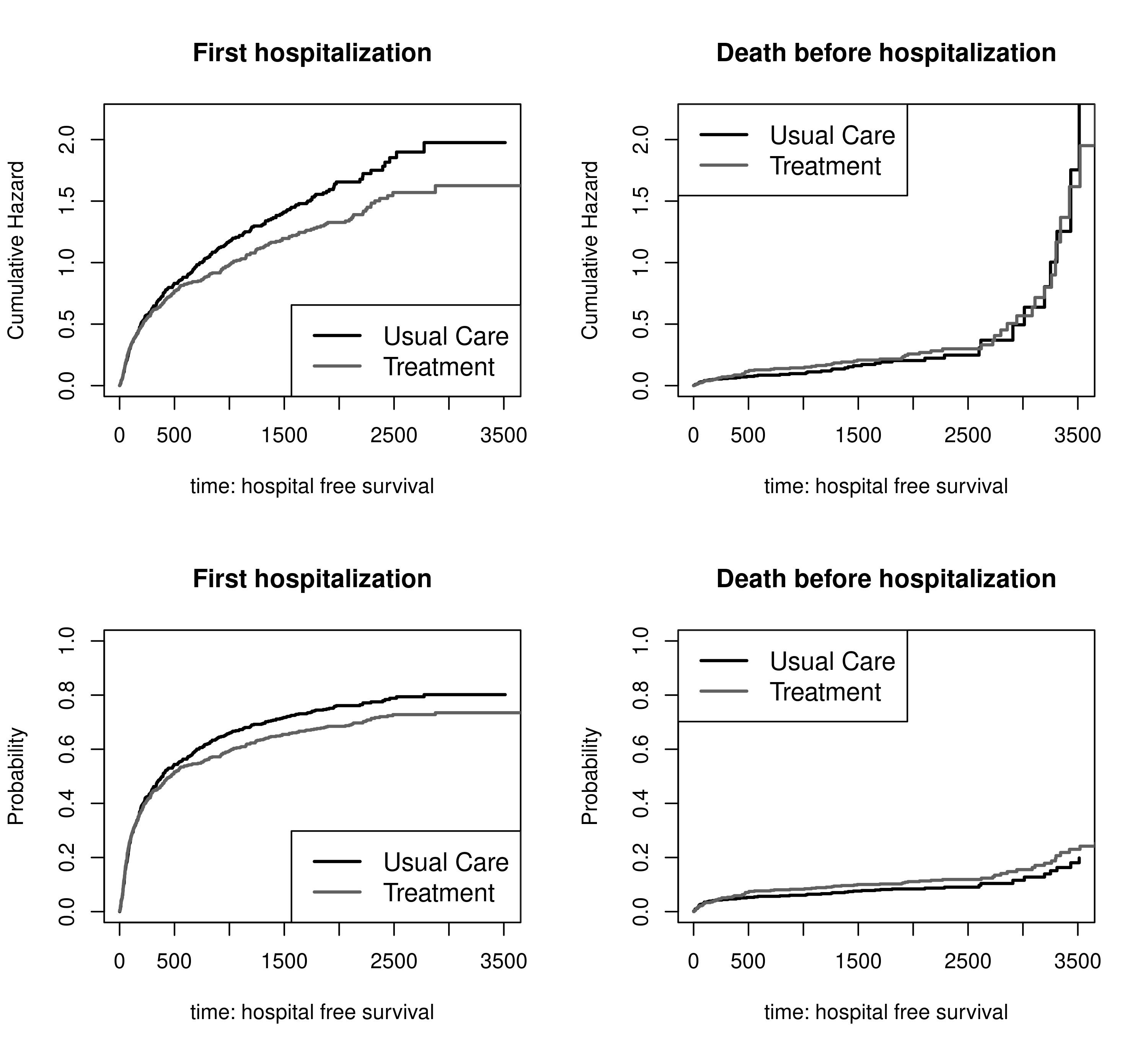

Supplement: Supplementary file 2 — Supporting File 2: bimj70107‐sup‐0001‐SuppMat.pdf. [file BIMJ-68-e70107-s002.zip › R_Files/Results_Study/Fig3_Main_CR.png]

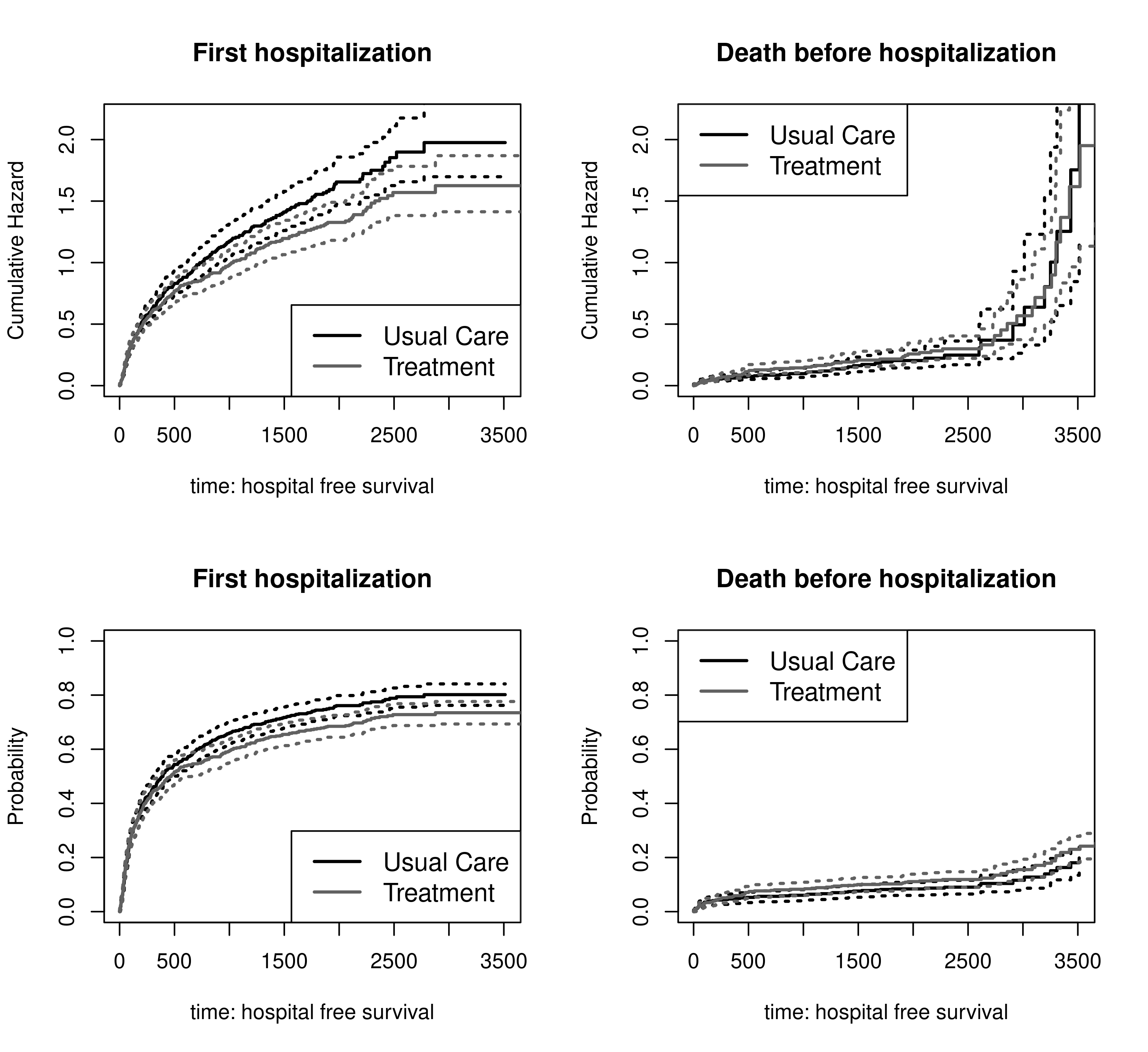

Supplement: Supplementary file 2 — Supporting File 2: bimj70107‐sup‐0001‐SuppMat.pdf. [file BIMJ-68-e70107-s002.zip › R_Files/Results_Study/Fig3_App_AppCR.png]

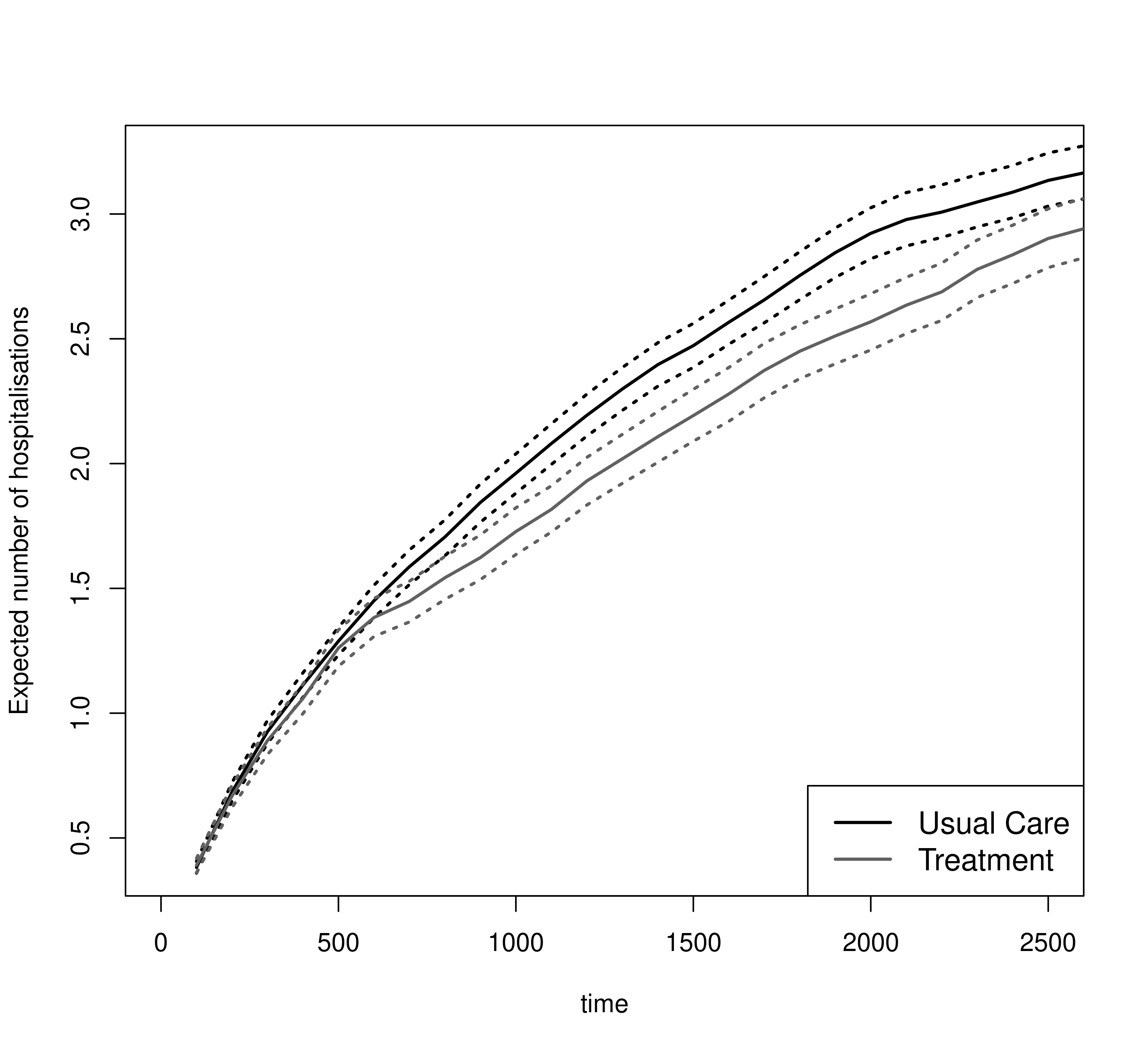

Supplement: Supplementary file 2 — Supporting File 2: bimj70107‐sup‐0001‐SuppMat.pdf. [file BIMJ-68-e70107-s002.zip › R_Files/Results_Study/Fig6_Main_Mean.png]

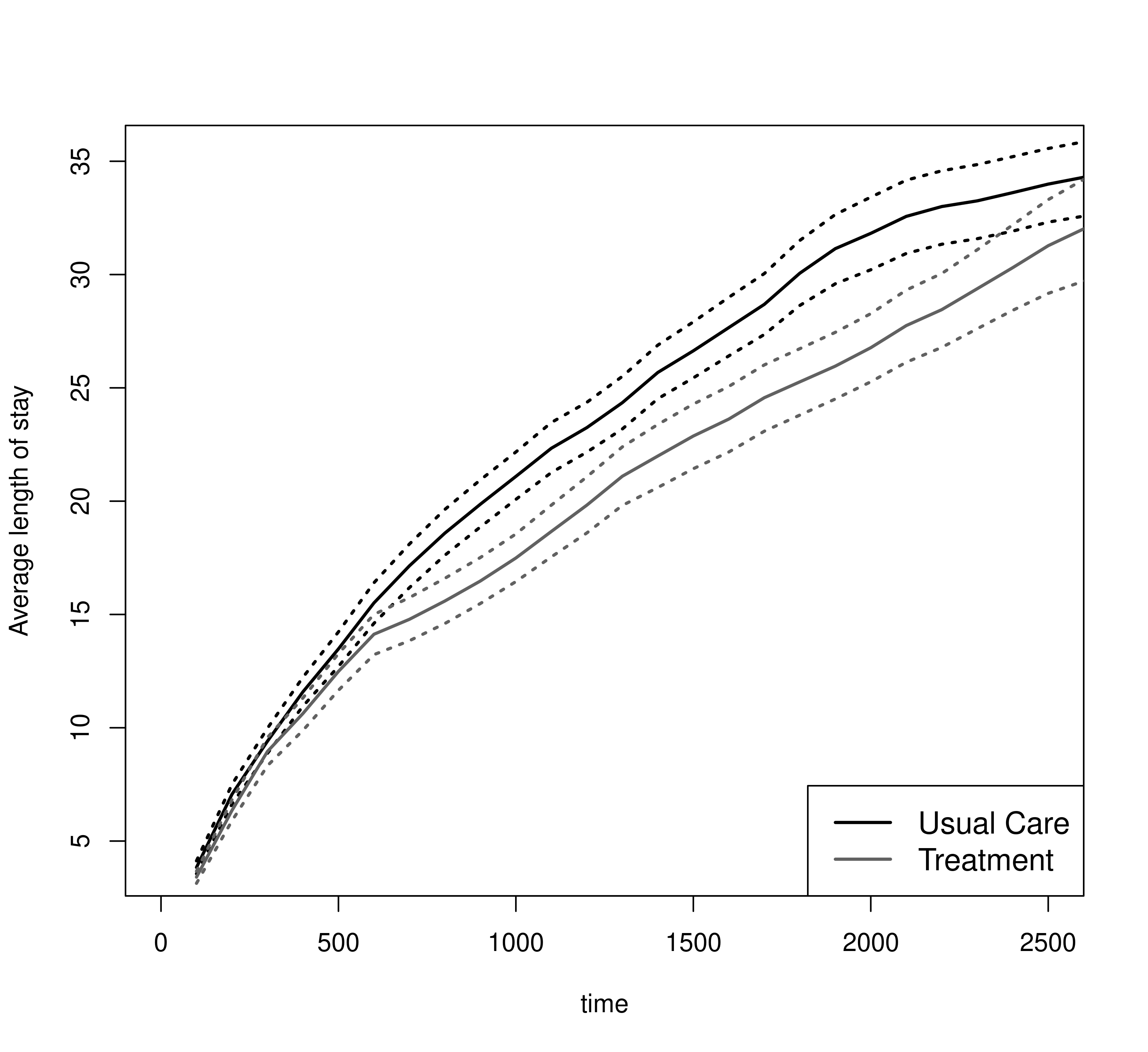

Supplement: Supplementary file 2 — Supporting File 2: bimj70107‐sup‐0001‐SuppMat.pdf. [file BIMJ-68-e70107-s002.zip › R_Files/Results_Study/Fig7_Main_Average.png]

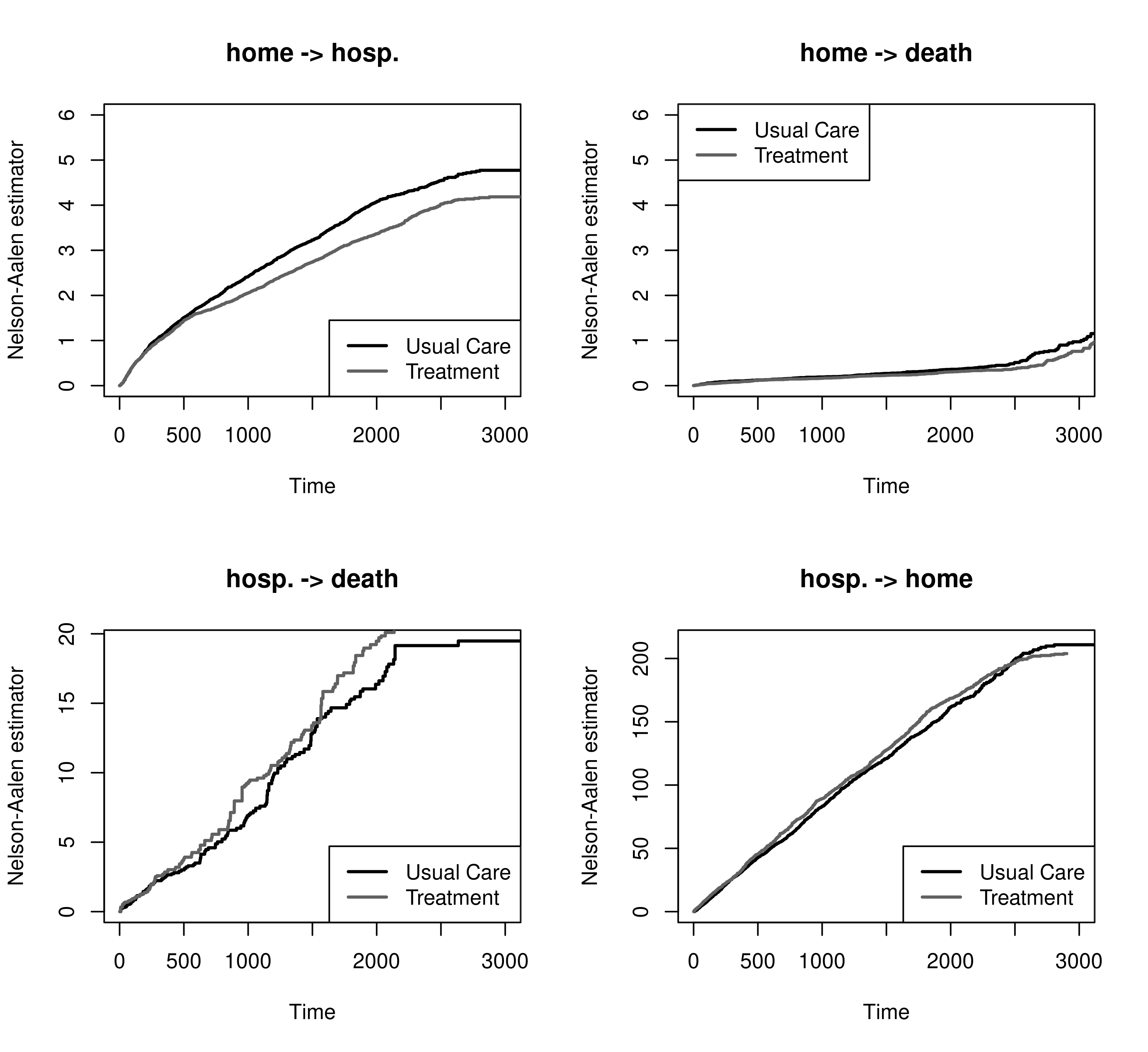

Supplement: Supplementary file 2 — Supporting File 2: bimj70107‐sup‐0001‐SuppMat.pdf. [file BIMJ-68-e70107-s002.zip › R_Files/Results_Study/Fig4_Main_Illdeath.png]

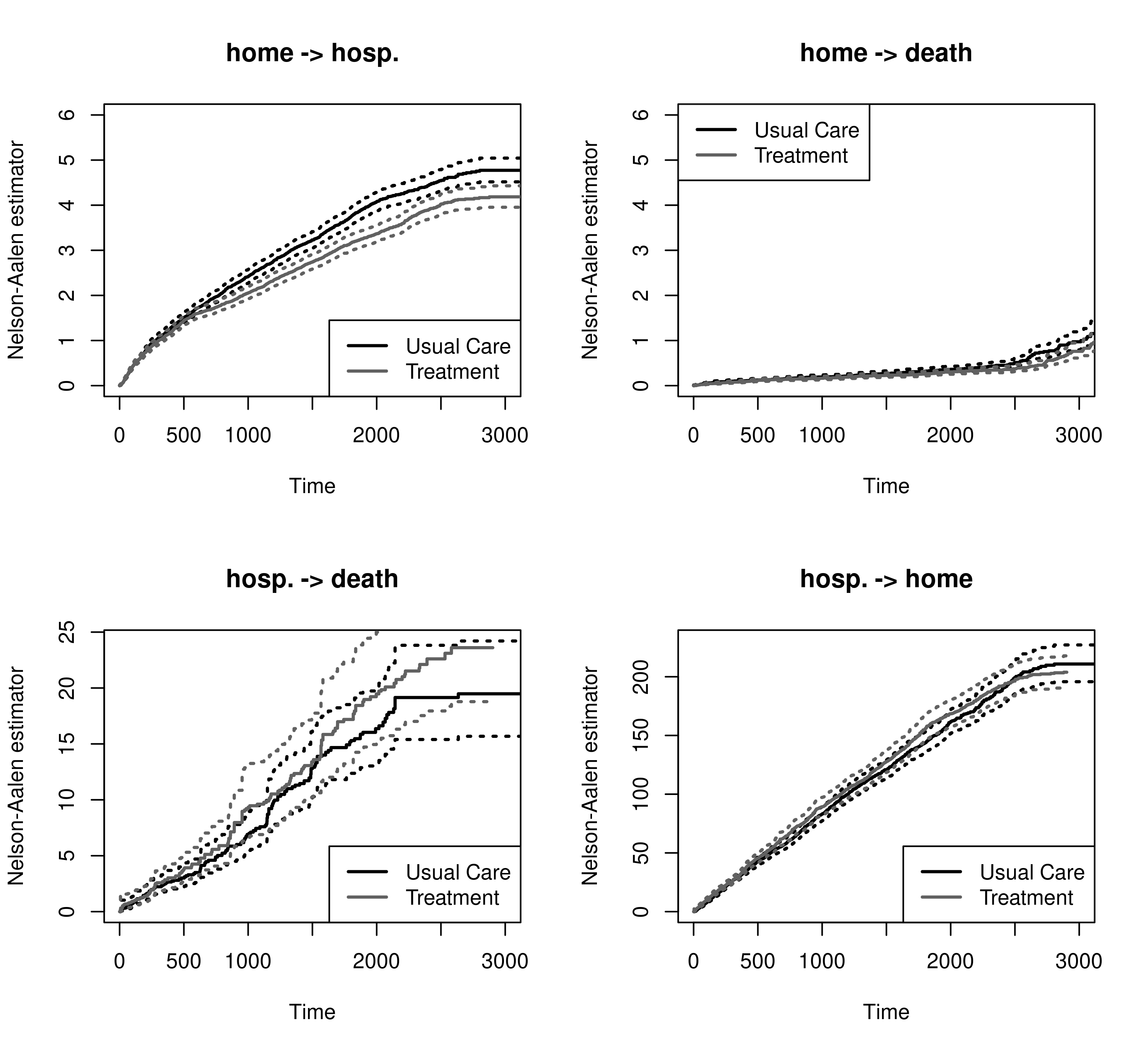

Supplement: Supplementary file 2 — Supporting File 2: bimj70107‐sup‐0001‐SuppMat.pdf. [file BIMJ-68-e70107-s002.zip › R_Files/Results_Study/Fig4_App_AppIlldeath.png]

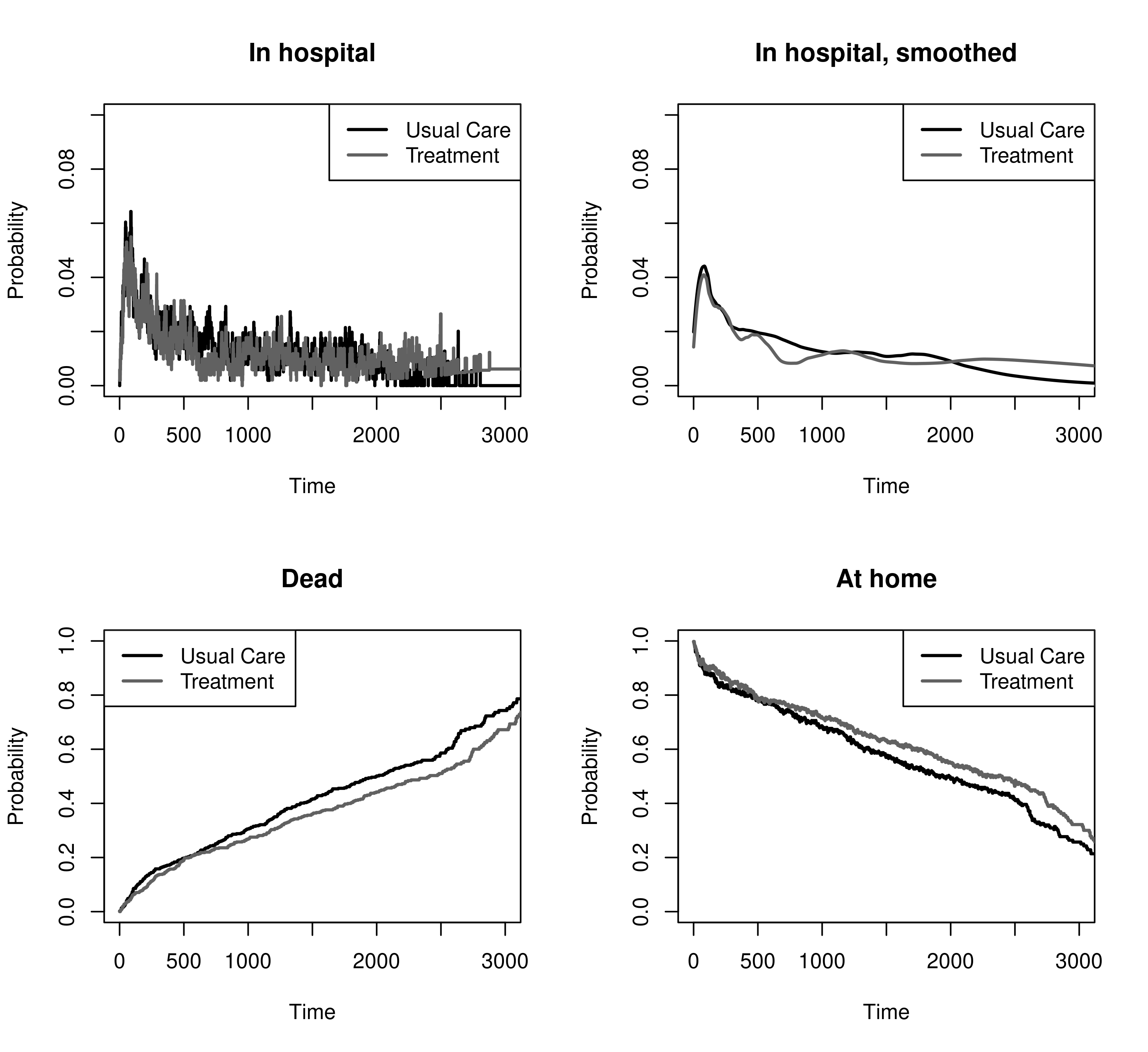

Supplement: Supplementary file 2 — Supporting File 2: bimj70107‐sup‐0001‐SuppMat.pdf. [file BIMJ-68-e70107-s002.zip › R_Files/Results_Study/Fig5_Main_State.png]

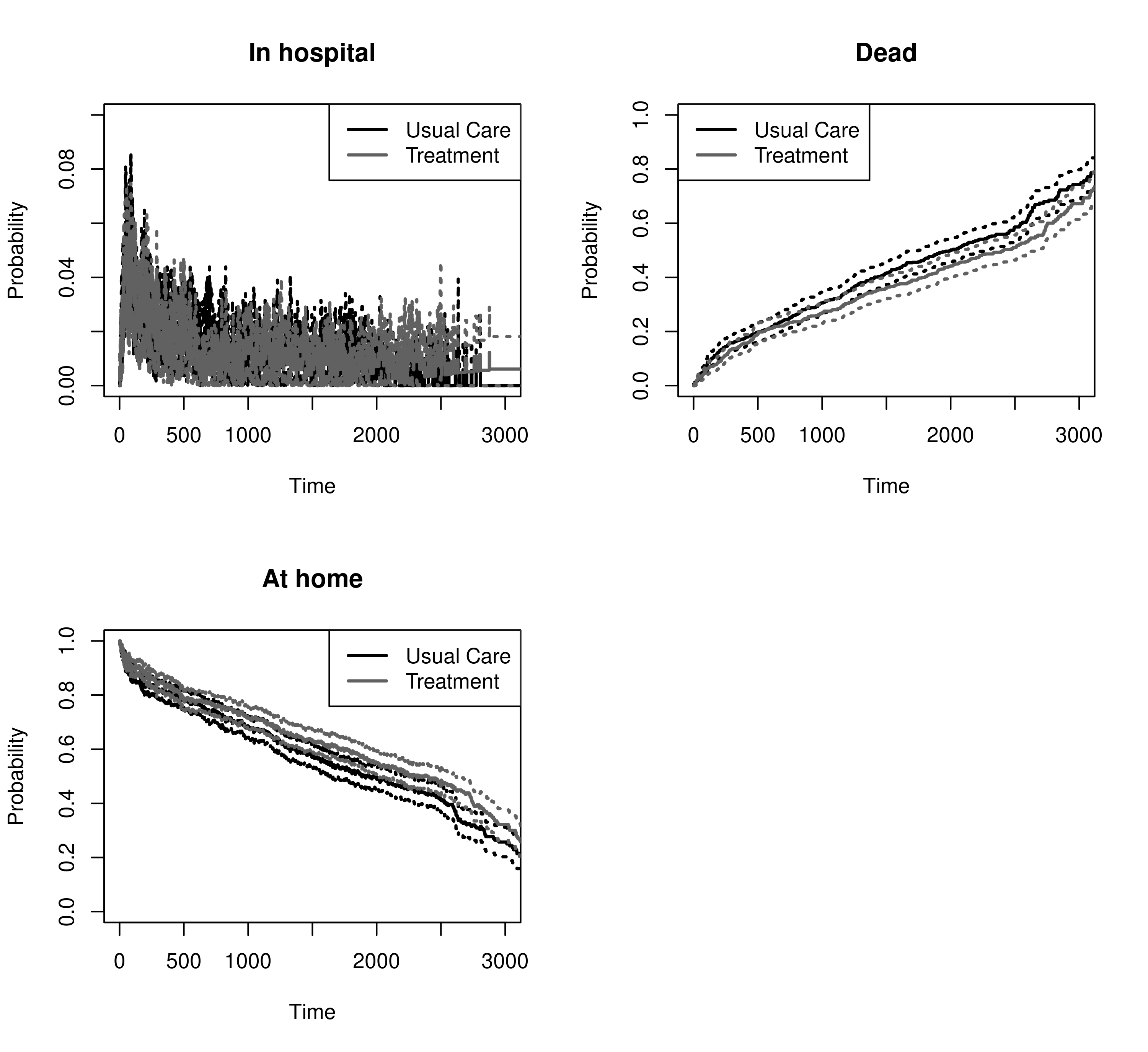

Supplement: Supplementary file 2 — Supporting File 2: bimj70107‐sup‐0001‐SuppMat.pdf. [file BIMJ-68-e70107-s002.zip › R_Files/Results_Study/Fig5_App_AppState.png]

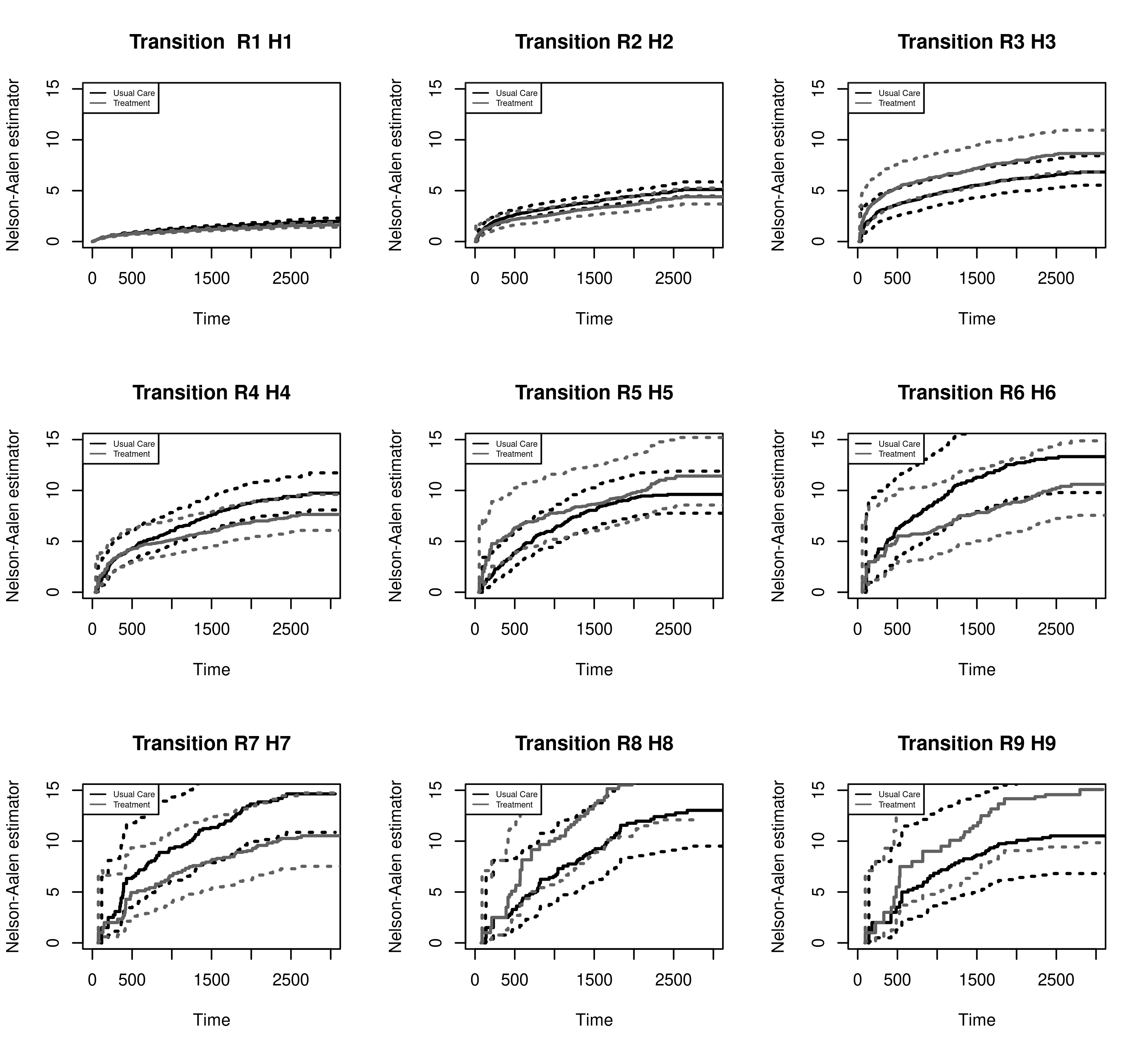

Supplement: Supplementary file 2 — Supporting File 2: bimj70107‐sup‐0001‐SuppMat.pdf. [file BIMJ-68-e70107-s002.zip › R_Files/Results_Study/Fig6_App_MSMNelAalHOSPApp.png]

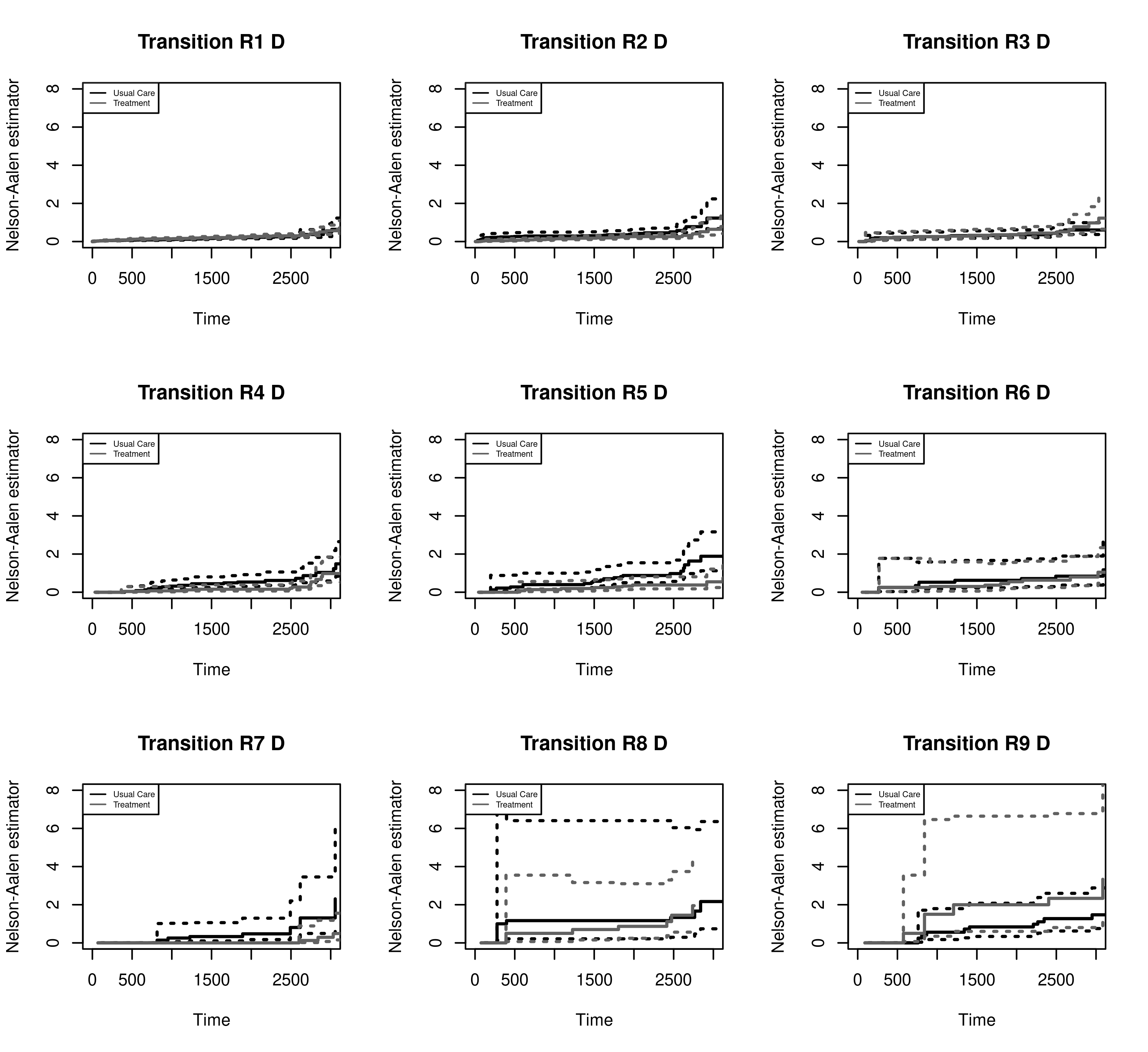

Supplement: Supplementary file 2 — Supporting File 2: bimj70107‐sup‐0001‐SuppMat.pdf. [file BIMJ-68-e70107-s002.zip › R_Files/Results_Study/Fig7_App_MSMNelAalDeathatHomeApp.png]

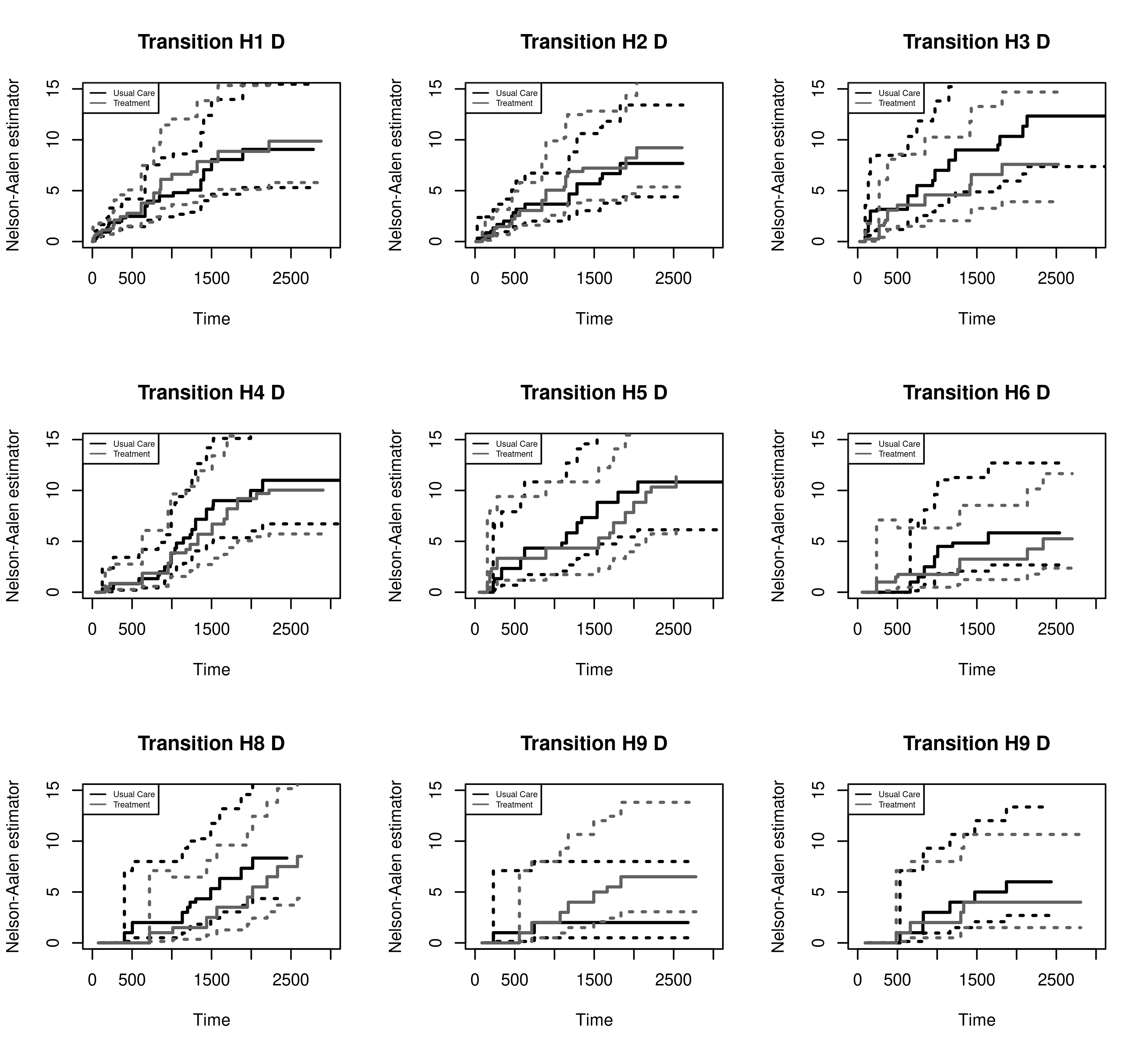

Supplement: Supplementary file 2 — Supporting File 2: bimj70107‐sup‐0001‐SuppMat.pdf. [file BIMJ-68-e70107-s002.zip › R_Files/Results_Study/Fig8_App_MSMNelAalDeathatHospitalApp.png]

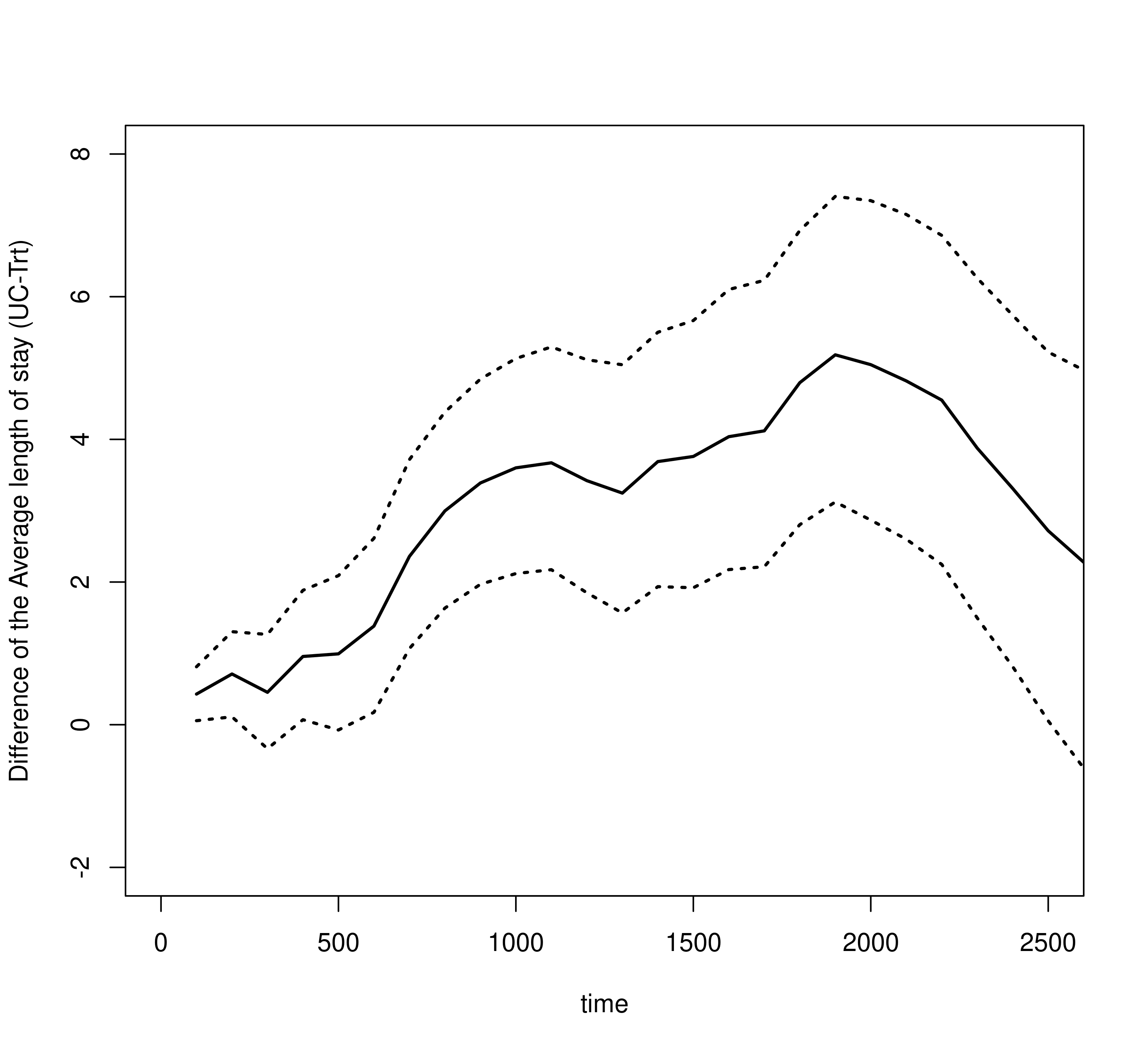

Supplement: Supplementary file 2 — Supporting File 2: bimj70107‐sup‐0001‐SuppMat.pdf. [file BIMJ-68-e70107-s002.zip › R_Files/Results_Study/Fig8_Main_AverageDIFF.png]

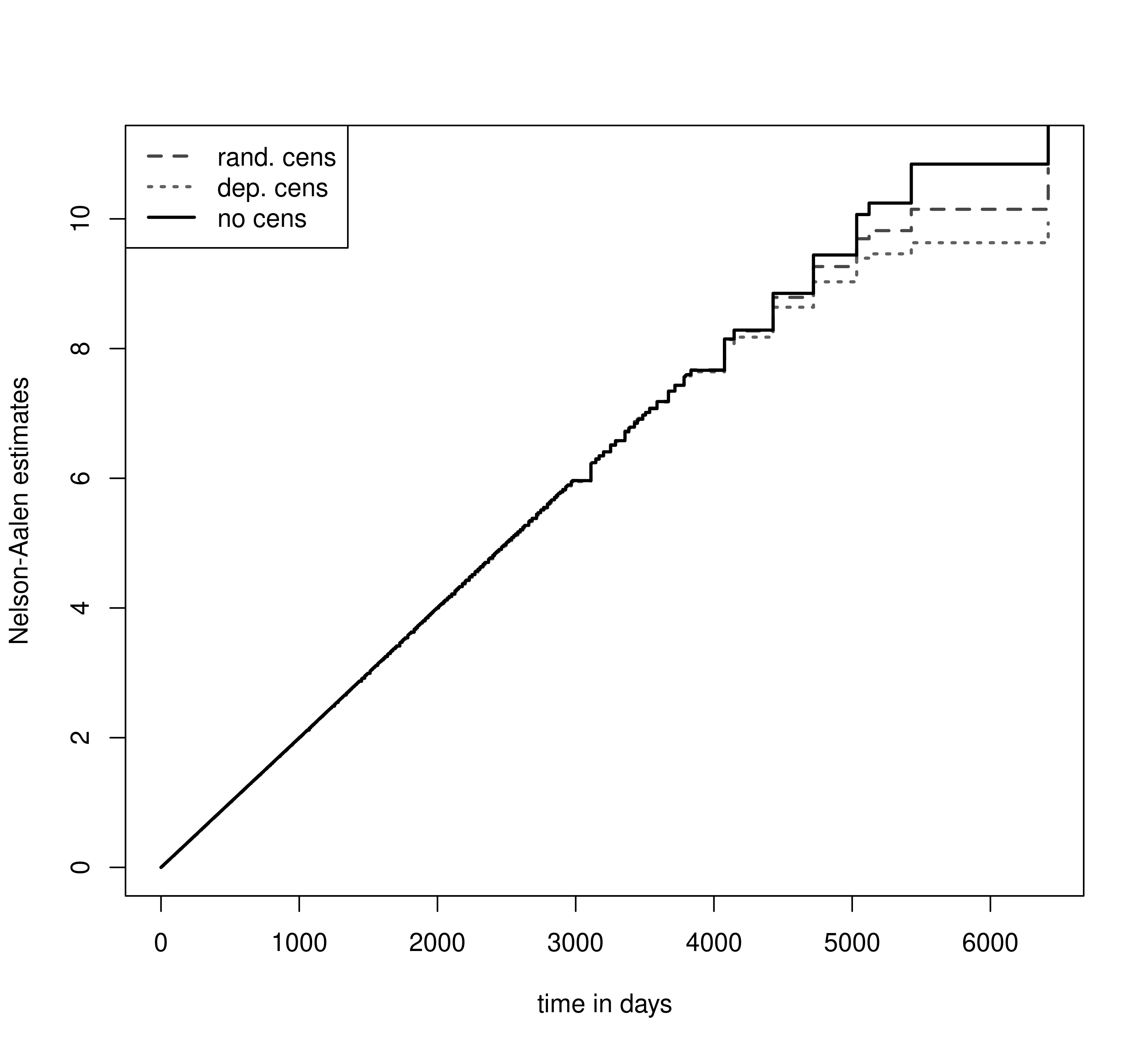

Supplement: Supplementary file 2 — Supporting File 2: bimj70107‐sup‐0001‐SuppMat.pdf. [file BIMJ-68-e70107-s002.zip › R_Files/Results_Simulation/Fig9_Main_SimNelAaMarkov.png]

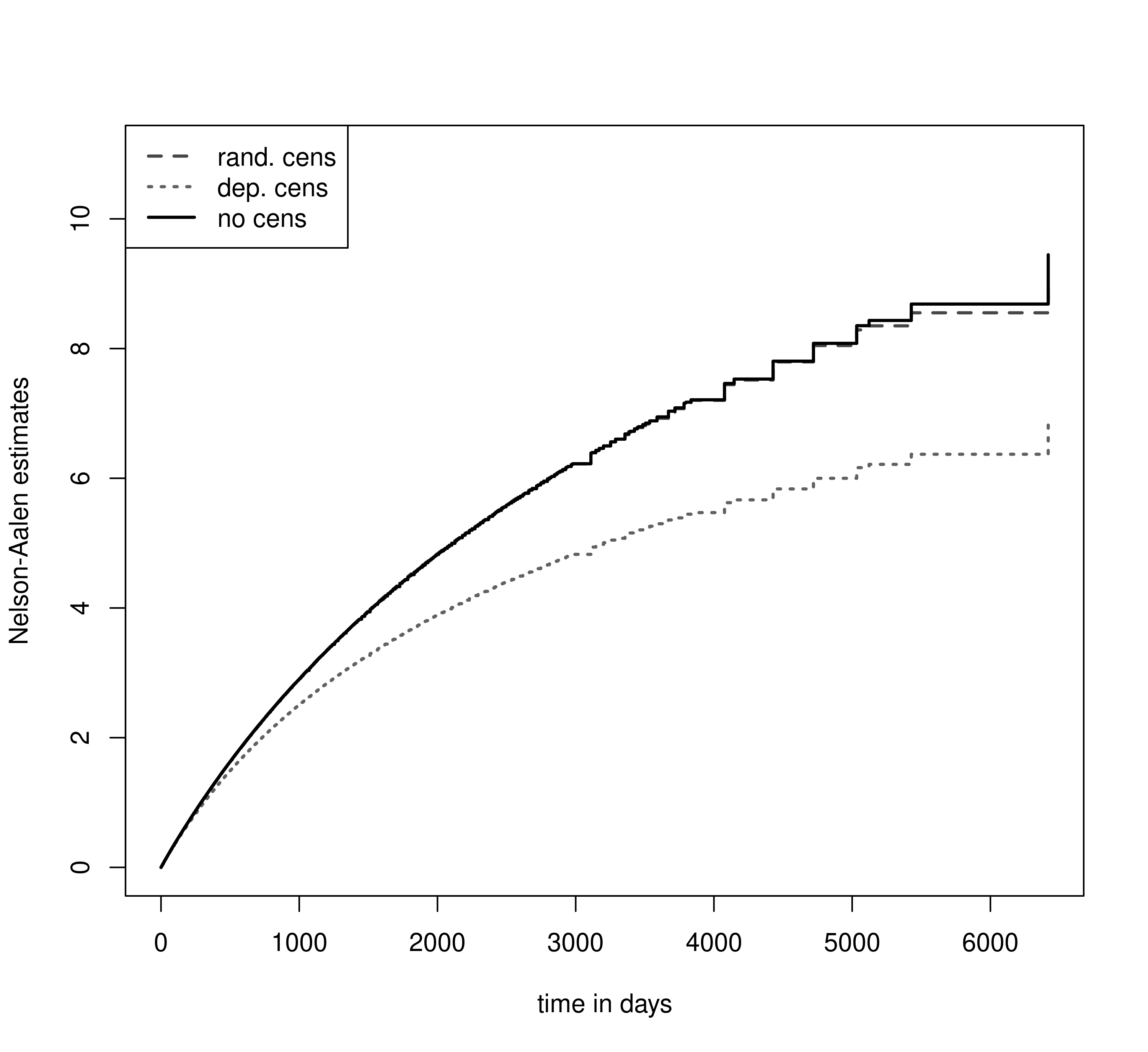

Supplement: Supplementary file 2 — Supporting File 2: bimj70107‐sup‐0001‐SuppMat.pdf. [file BIMJ-68-e70107-s002.zip › R_Files/Results_Simulation/Fig10_Main_SimNelAaNONMarkov.png]

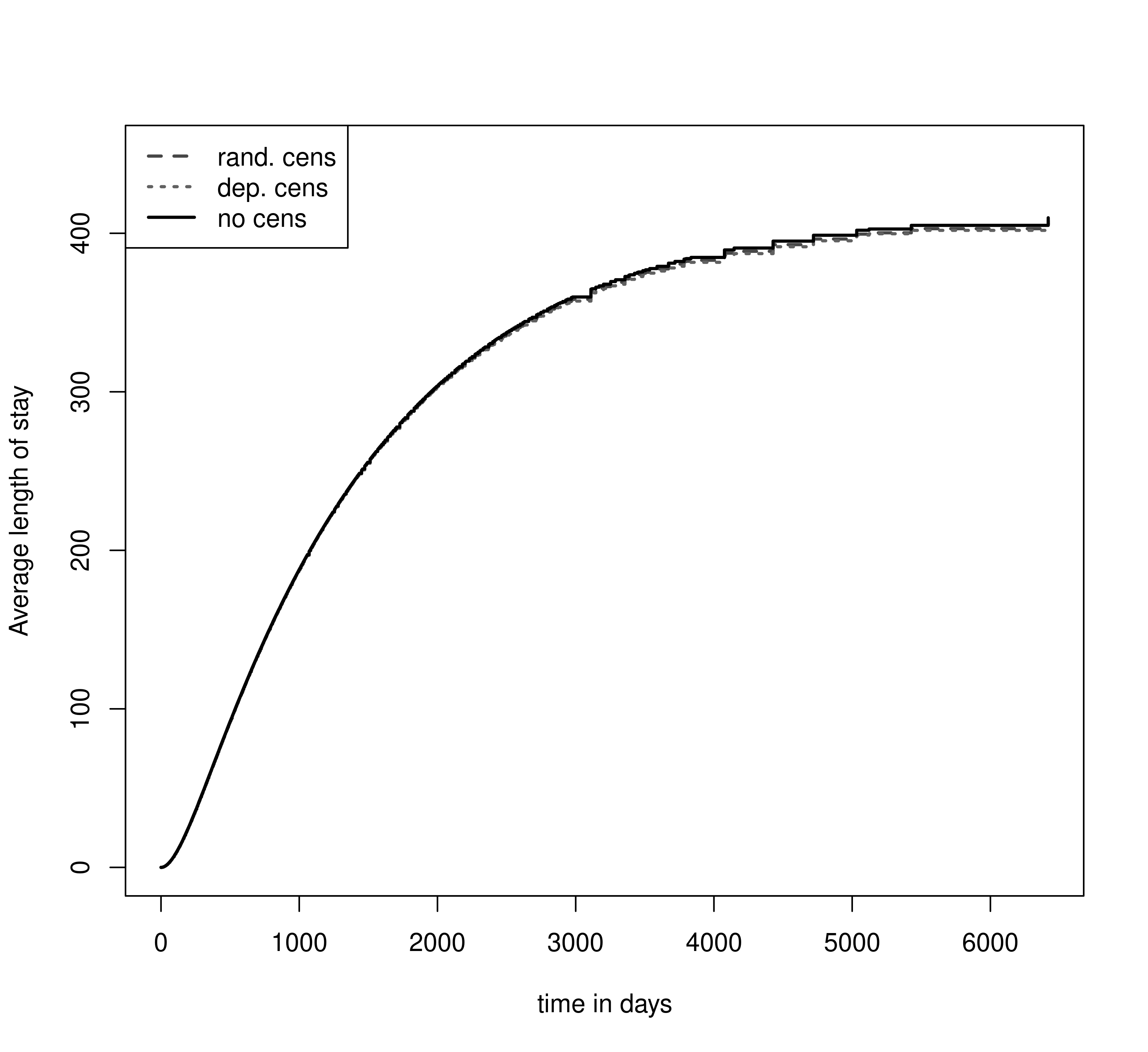

Supplement: Supplementary file 2 — Supporting File 2: bimj70107‐sup‐0001‐SuppMat.pdf. [file BIMJ-68-e70107-s002.zip › R_Files/Results_Simulation/Fig9_App_SimLengthMarkov.png]

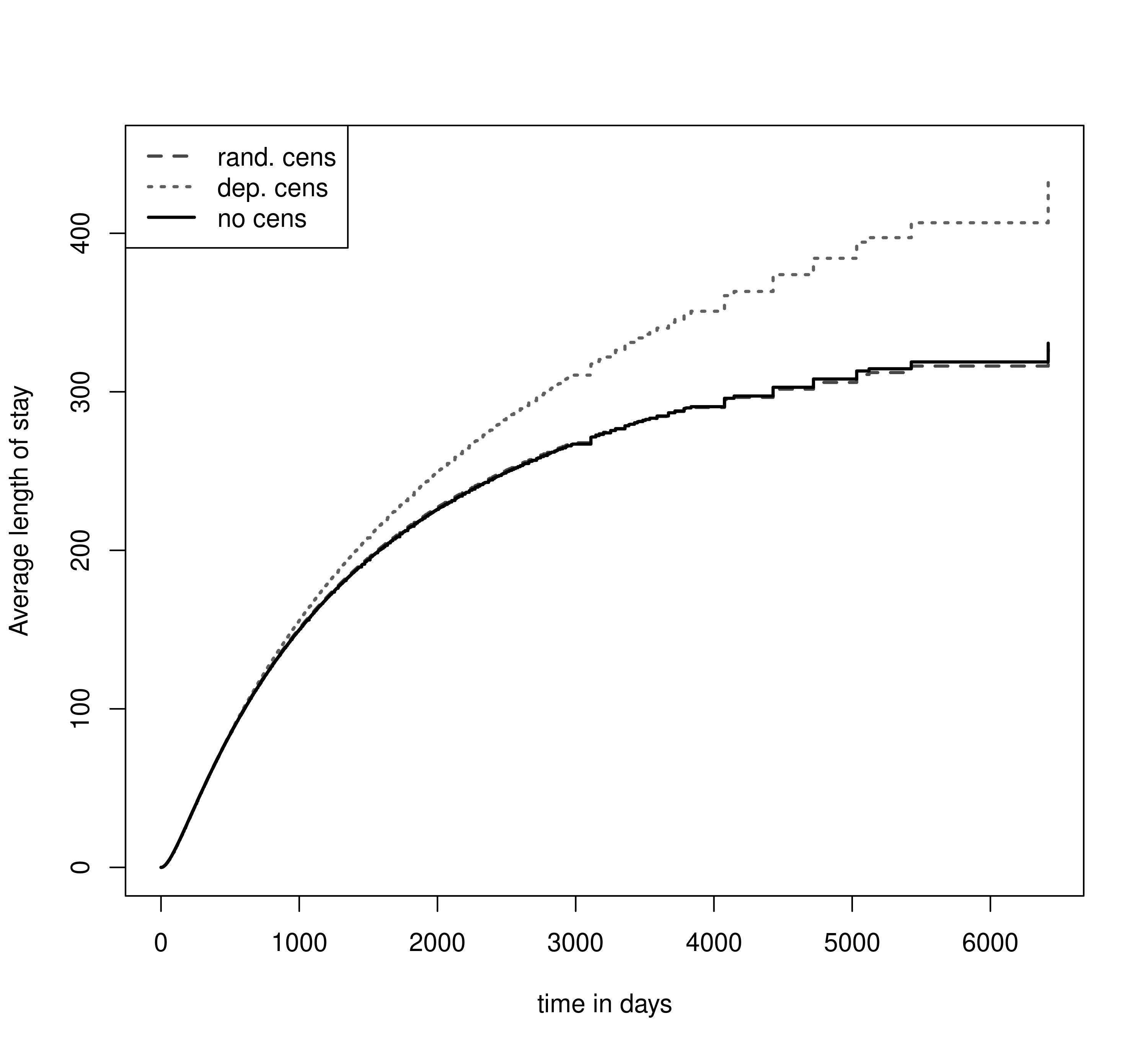

Supplement: Supplementary file 2 — Supporting File 2: bimj70107‐sup‐0001‐SuppMat.pdf. [file BIMJ-68-e70107-s002.zip › R_Files/Results_Simulation/Fig10_App_SimLengthNONMarkov.png]

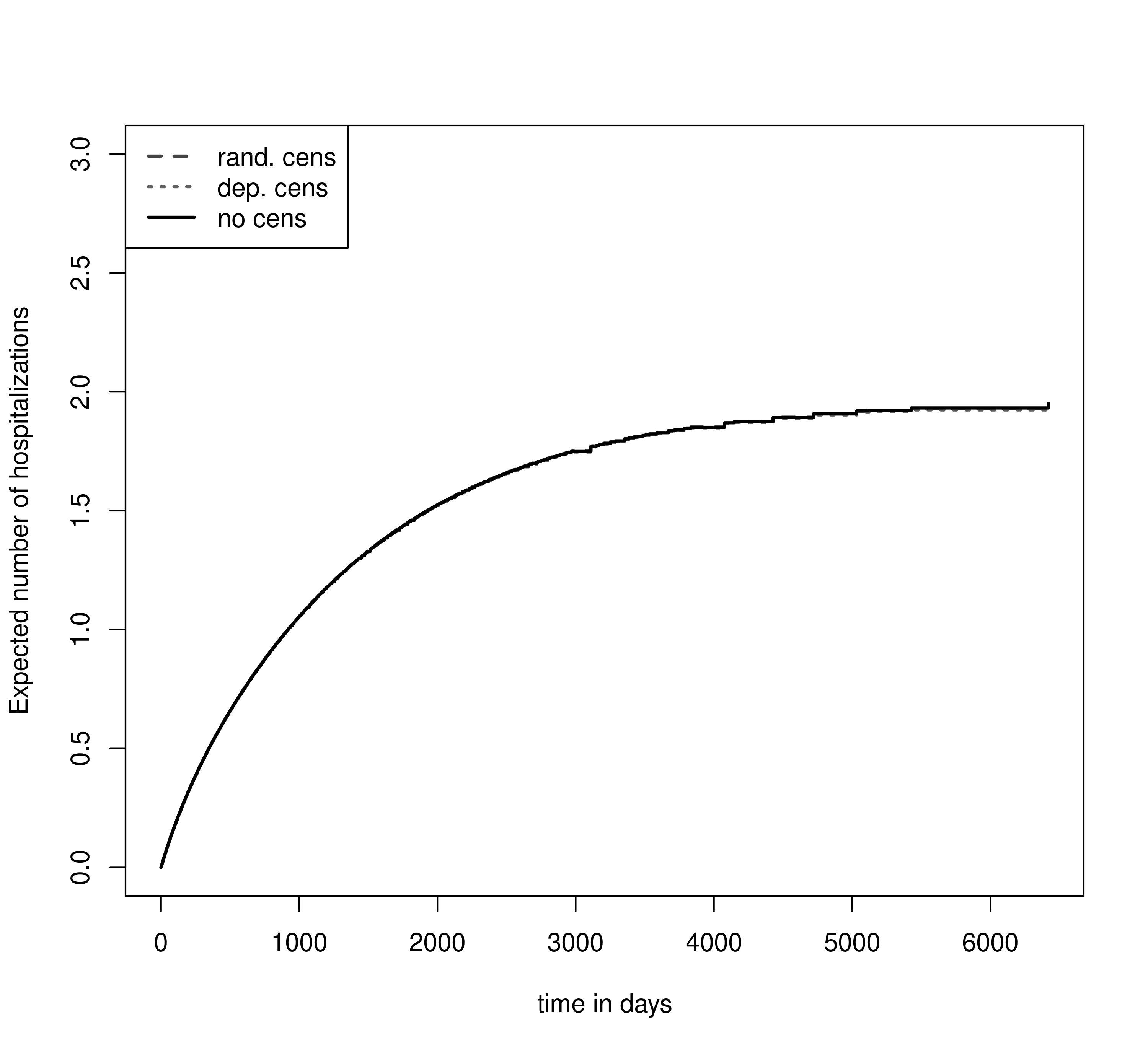

Supplement: Supplementary file 2 — Supporting File 2: bimj70107‐sup‐0001‐SuppMat.pdf. [file BIMJ-68-e70107-s002.zip › R_Files/Results_Simulation/Fig11_App_SimMeanMarkov.png]

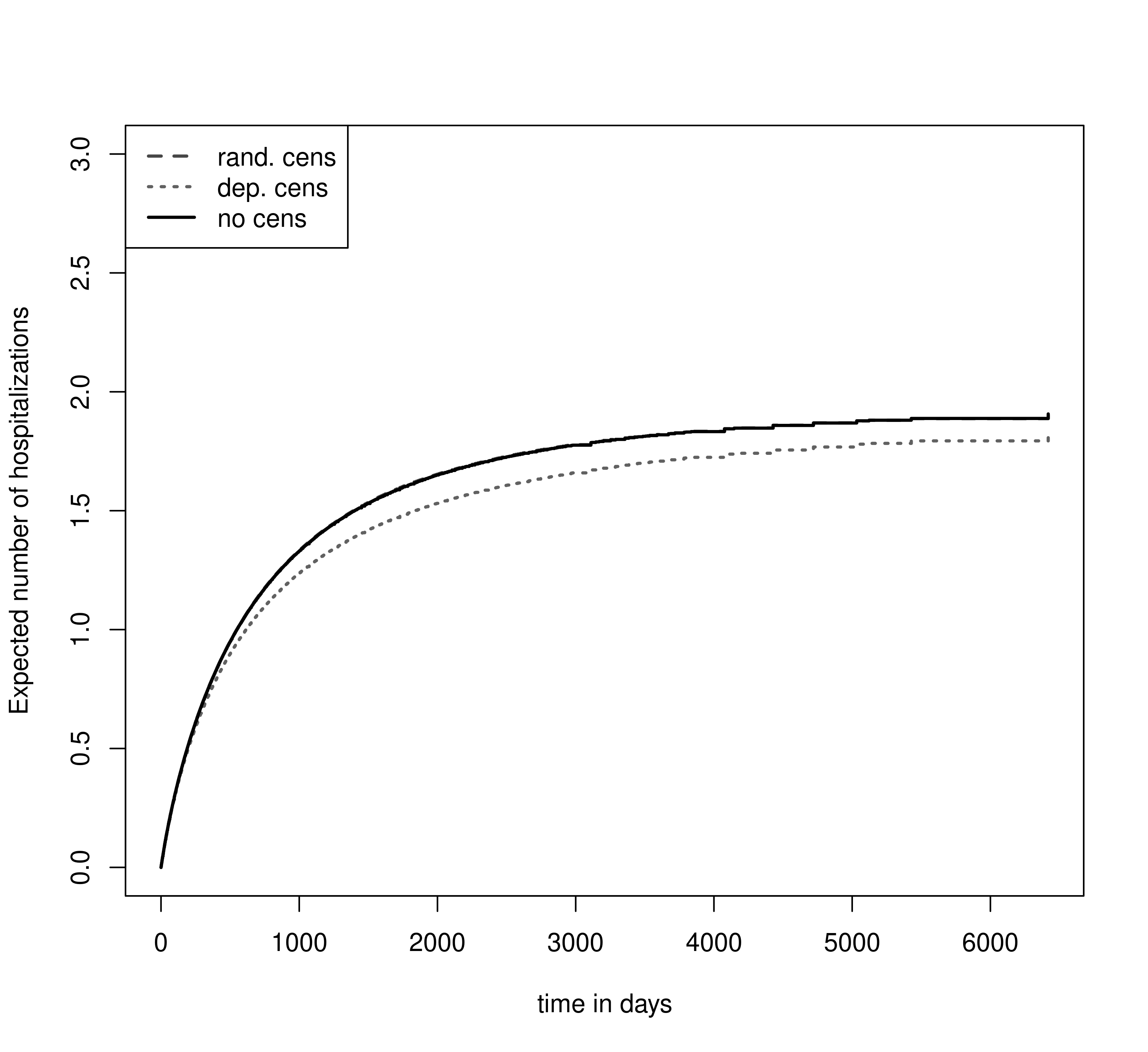

Supplement: Supplementary file 2 — Supporting File 2: bimj70107‐sup‐0001‐SuppMat.pdf. [file BIMJ-68-e70107-s002.zip › R_Files/Results_Simulation/Fig12_App_SimMeanNONMarkov.png]

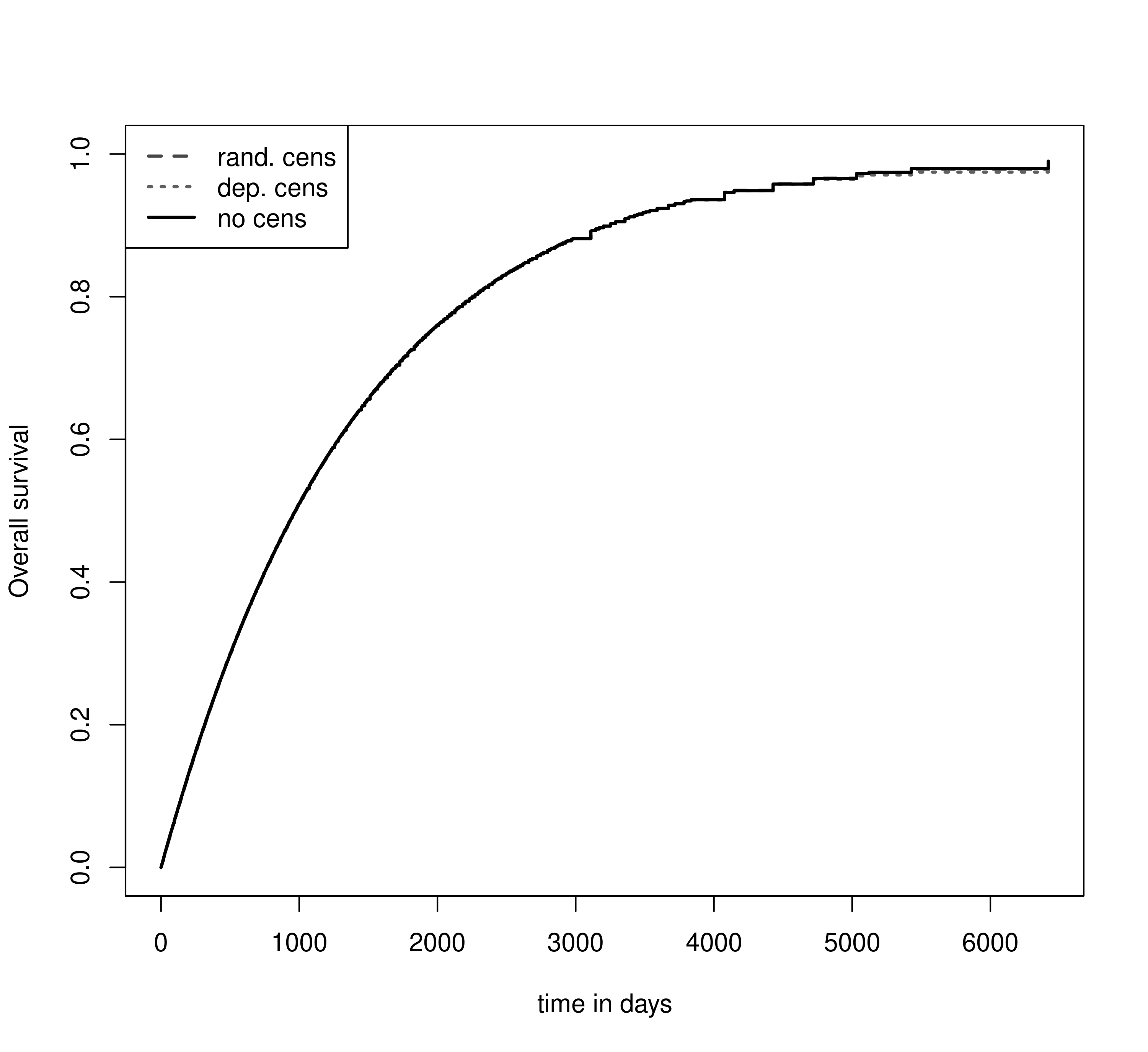

Supplement: Supplementary file 2 — Supporting File 2: bimj70107‐sup‐0001‐SuppMat.pdf. [file BIMJ-68-e70107-s002.zip › R_Files/Results_Simulation/Fig13_App_SimOSMarkov.png]

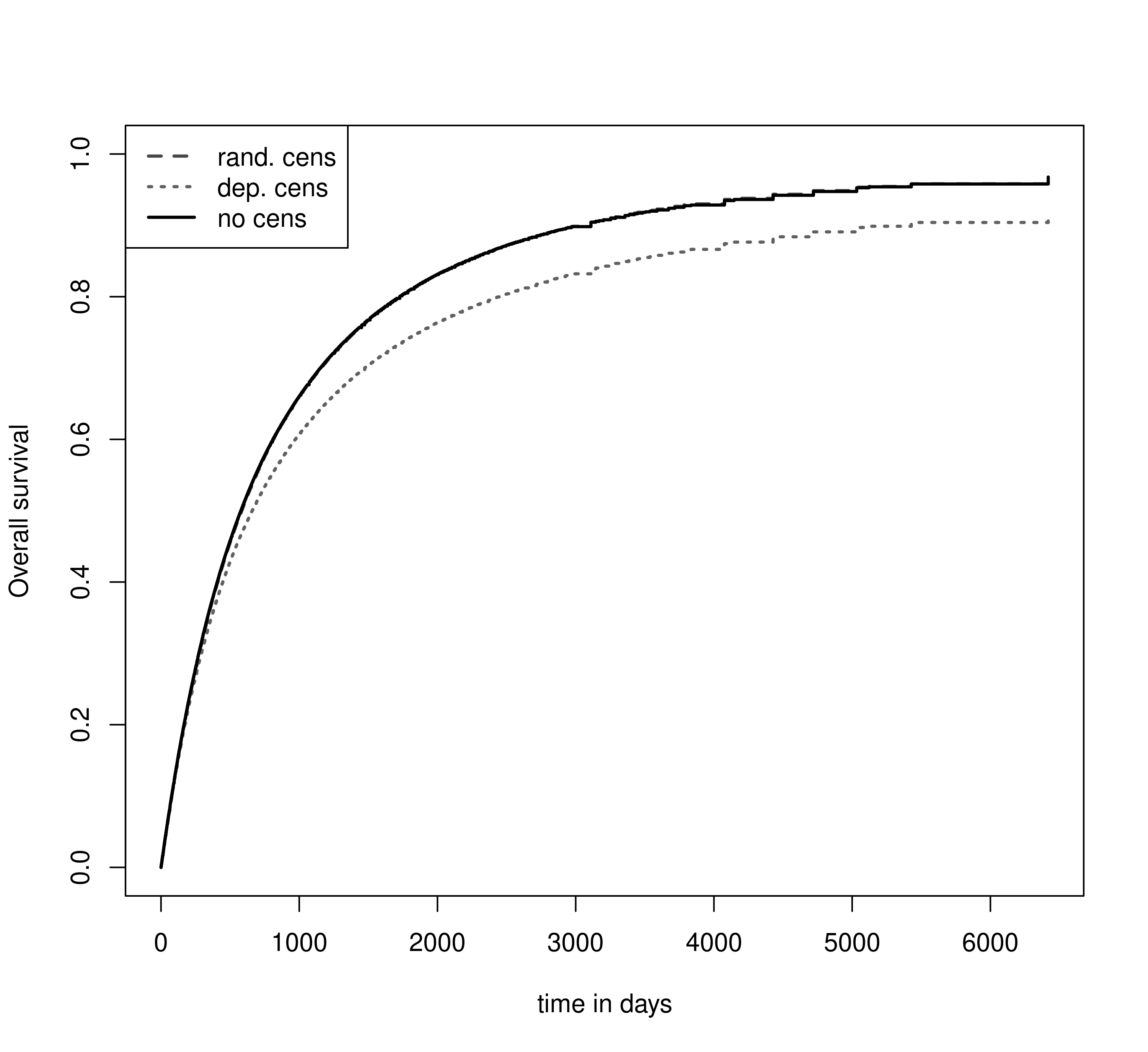

Supplement: Supplementary file 2 — Supporting File 2: bimj70107‐sup‐0001‐SuppMat.pdf. [file BIMJ-68-e70107-s002.zip › R_Files/Results_Simulation/Fig14_App_SimOSNONMarkov.png]
